# Crosstalk Between Autophagy and Oxidative Stress in Hematological Malignancies: Mechanisms, Implications, and Therapeutic Potential

**DOI:** 10.3390/antiox14030264

**Published:** 2025-02-25

**Authors:** Antonio José Cabrera-Serrano, José Manuel Sánchez-Maldonado, Carmen González-Olmedo, María Carretero-Fernández, Leticia Díaz-Beltrán, Juan Francisco Gutiérrez-Bautista, Francisco José García-Verdejo, Fernando Gálvez-Montosa, José Antonio López-López, Paloma García-Martín, Eva María Pérez, Pedro Sánchez-Rovira, Fernando Jesús Reyes-Zurita, Juan Sainz

**Affiliations:** 1 Genomic Oncology Area, GENYO, Centre for Genomics and Oncological Research: Pfizer/University of Granada/Andalusian Regional Government, PTS, 18016 Granada, Spain; antonio.cabrera@genyo.es (A.J.C.-S.); josemanuel.sanchez@genyo.es (J.M.S.-M.); cgonzalezolmedo@fibao.es (C.G.-O.); maria.carretero@genyo.es (M.C.-F.); ldiaz@fibao.es (L.D.-B.); juanfrety@gmail.com (J.F.G.-B.); fjgverdejo@gmail.com (F.J.G.-V.); fernando.galvez.sspa@juntadeandalucia.es (F.G.-M.); josea.lopez.l.sspa@juntadeandalucia.es (J.A.L.-L.); evam.perez.sspa@juntadeandalucia.es (E.M.P.); pedro.sanchez.rovira.sspa@juntadeandalucia.es (P.S.-R.); 2 Instituto de Investigación Biosanitaria IBs.Granada, 18012 Granada, Spain; paloma.garcia.martin.sspa@juntadeandalucia.es; 3 Department of Biochemistry and Molecular Biology I, Faculty of Sciences, University of Granada, 18012 Granada, Spain; 4 Medical Oncology Unit, University Hospital of Jaén, 23007 Jaén, Spain; 5 Servicio de Análisis Clínicos e Inmunología, University Hospital Virgen de las Nieves, 18014 Granada, Spain; 6 Department of Biochemistry, Molecular Biology and Immunology III, University of Granada, 18016 Granada, Spain; 7 Campus de la Salud Hospital, PTS, 18016 Granada, Spain; 8 CIBER Epidemiología y Salud Pública (CIBERESP), 28029 Madrid, Spain

**Keywords:** autophagy, oxidative stress, reactive oxygen species, crosstalk, hematological malignancies, cancer treatment outcomes, therapeutic opportunities

## Abstract

Autophagy is a fundamental cellular process that maintains homeostasis by degrading damaged components and regulating stress responses. It plays a crucial role in cancer biology, including tumor progression, metastasis, and therapeutic resistance. Oxidative stress, similarly, is key to maintaining cellular balance by regulating oxidants and antioxidants, with its disruption leading to molecular damage. The interplay between autophagy and oxidative stress is particularly significant, as reactive oxygen species (ROS) act as both inducers and by-products of autophagy. While autophagy can function as a tumor suppressor in early cancer stages, it often shifts to a pro-tumorigenic role in advanced disease, aiding cancer cell survival under adverse conditions such as hypoxia and nutrient deprivation. This dual role is mediated by several signaling pathways, including PI3K/AKT/mTOR, AMPK, and HIF-1α, which coordinate the balance between autophagic activity and ROS production. In this review, we explore the mechanisms by which autophagy and oxidative stress interact across different hematological malignancies. We discuss how oxidative stress triggers autophagy, creating a feedback loop that promotes tumor survival, and how autophagic dysregulation leads to increased ROS accumulation, exacerbating tumorigenesis. We also examine the therapeutic implications of targeting the autophagy–oxidative stress axis in cancer. Current strategies involve modulating autophagy through specific inhibitors, enhancing ROS levels with pro-oxidant compounds, and combining these approaches with conventional therapies to overcome drug resistance. Understanding the complex relationship between autophagy and oxidative stress provides critical insights into novel therapeutic strategies aimed at improving cancer treatment outcomes.

## 1. Introduction

Cancer represents a significant challenge in modern society, posing substantial public health and economic burdens in the 21st century. Globally, it accounts for nearly one in six deaths (16.8%) and approximately one in four deaths (22.8%) attributed to non-communicable diseases. Furthermore, cancer is responsible for 30.3% of premature deaths from non-communicable diseases among individuals aged 30–69 years, making it one of the three leading causes of mortality in this age group in 177 out of 183 countries [1]. A recent study based on the 2022 GLOBOCAN estimates highlighted significant geographic variability in cancer incidence and mortality across 20 world regions, focusing on the 10 most common cancer types (https://gco.iarc.who.int/today, 20 November 2024). It further explored new opportunities for global cancer prevention and control and underscored the critical need for new targeted prevention strategies.

Given the complexity and heterogeneity of tumors, autophagy and oxidative stress have emerged in recent years as critical cellular processes in cancer development and progression. These mechanisms play pivotal roles in human health. Autophagy is an essential mechanism for maintaining cellular homeostasis [2,3], for preventing metabolic imbalance and the accumulation of cytotoxic elements within cells, and for prolonging cell survival [4,5,6,7,8]. On the other hand, oxidative stress arises from an imbalance between the production of reactive oxygen species (ROS) and the effectiveness of cellular antioxidant defenses. This imbalance often leads to DNA damage, genomic instability, cellular dysfunction, and disease [3,9]. While autophagy can limit oxidative stress by degrading damaged mitochondria (mitophagy), excessive levels of ROS can trigger autophagic activity to promote cell survival under adverse conditions [3,10]. Autophagy and oxidative stress are deeply interconnected in biology, with their roles oscillating between protective and pathological depending on the context [11]. This review explores the intricate relationship between autophagy and oxidative stress, examining their dual roles in tumorigenesis, their implications for therapeutic intervention, and potential future research directions to further elucidate these complex processes that may exhibit a synergistic and dual role, acting as both tumor suppressors and promoters depending on the specific cellular and microenvironmental context [12,13,14,15].

## 2. Mechanisms of Autophagy

Autophagy, a fundamental catabolic process in cellular homeostasis, acts in close coordination with other crucial mechanisms of cellular control, such as apoptosis and the proteasome system, to maintain cellular integrity and function [16]. This highly regulated pathway unfolds through a series of well-orchestrated steps, each mediated by a complex network of genes and proteins. Among the most extensively studied are the autophagy-related genes (ATG), which play a pivotal role in the autophagy process [17]. Additionally, key regulators such as the mechanistic target of rapamycin complex 1 (mTORC1), a serine/threonine kinase, and the phosphatidylinositol 3-kinase (PI3K) complex are critically involved in modulating this pathway [18,19,20].

The autophagic process is typically divided into distinct stages, including initiation, nucleation, elongation, lysosome fusion, and autophagosome degradation [17]. Each step is characterized by specific molecular events and regulatory mechanisms that ensure the efficient turnover of cellular components and adaptation to stress conditions [20,21]. Understanding these stages in detail is crucial for elucidating the role of autophagy in health and disease, as well as for identifying potential therapeutic targets in pathological contexts.

### 2.1. Molecular Machinery and Signaling Pathways

#### 2.1.1. Initiation

Autophagy initiation begins with the formation of the autophagosome, requiring the synthesis of an isolation membrane, or “omegasome”, which originates from the ER. This membrane develops into the phagophore, a cup-based structure composed of a single membrane. While the ER is the primary source, other organelles like the Golgi apparatus, endosomes, mitochondria, and plasma membrane also contribute to its formation [21]. mTORC1 regulates this stage, especially under nutrient deprivation. mTORC1 exists in two functionally distinct complexes: the rapamycin-sensitive mTORC1 that regulates cell size, and mTORC2 that is involved in modulating actin cytoskeleton organization. When nutrients are plentiful, mTORC1 localizes to the lysosome, where it is activated by the Rheb subunit, suppressing autophagy. Rapamycin, an mTORC1 inhibitor, induces autophagy even in nutrient-rich conditions [22,23]. mTORC1’s regulation of autophagy initiation involves its interaction with the ULK1 complex, which consists of ULK1, ATG13, ATG101, and FIP200. Under nutrient-rich conditions, mTORC1 phosphorylates ULK1 and ATG13, inhibiting the ULK1 complex and autophagy. Under nutrient deprivation, mTORC1 is inhibited, releasing the ULK1 complex to activate autophagy through AMPK’s phosphorylation of Rheb and RAPTOR. The activated ULK1 complex then facilitates the formation of the phagophore by phosphorylating Beclin-1 within the PI3K complex, triggering autophagic pathway initiation [23,24,25,26].

#### 2.1.2. Nucleation and Elongation

Autophagosome nucleation is triggered by the Class III PI3K complex, composed of VPS34, Beclin-1 (ATG6), ATG14L, and p150 (VPS15) [27]. Beclin-1, located on the ER membrane, modulates this complex by binding to UVRAG or members of BCL2 family members, activating or inhibiting autophagy. ULK1, when activated, phosphorylates Beclin-1 and AMBRA1, promoting the PI3K complex recruitment to the ER and facilitating omegasome formation, the initial structure for the phagophore [24,28,29]. The activated PI3K in the ER produces phosphatidylinositol 3-phosphate (PIP3) on the omegasome membrane, recruiting WIPI proteins that attract other ATG proteins crucial for autophagy. ATG9, the only transmembrane ATG protein, is essential for lipid transport to the phagophore. The phagophore expands via two ubiquitin-like conjugation systems: the ATG12-ATG5-ATG16L system and LC3-II (ATG8). LC3 is conjugated with phosphatidylethanolamine, forming LC3-II, which becomes inserted into the expanding phagophore membrane and serves as an autophagosome marker, facilitating selective autophagy by interacting with autophagic cargo receptors [24,30,31].

#### 2.1.3. Selective Autophagy

Although autophagy is generally non-selective, evidence suggests substrate selectivity, as exemplified by LC3-II’s interaction with SQSTM1/p62. This adaptor protein binds ubiquitinated proteins, facilitating their capture and delivery to autophagosomes through LC3-II in a process termed LC3-associated phagocytosis. In addition, chaperone-mediated autophagy provides additional selectivity mechanisms, underscoring the dynamic adaptability of autophagy to several cellular needs [25,30,32,33].

#### 2.1.4. Fusion with Lysosomes and Degradation

Upon completion, the autophagosome fuses with endosomes via the HOPS complex and then with lysosomes to form an autolysosome. The Rab7 GTPase protein, activated by UVRAG, regulates this process, with SNARE proteins mediating membrane fusion. Proteins LAMP1 and LAMP2 stabilize the fusion process, facilitating material transport. The lysosomal enzymes then degrade the autophagosome’s contents, with permeases excreting the breakdown products into the cytosol for recycling [23,24,34].

**Figure 1:**
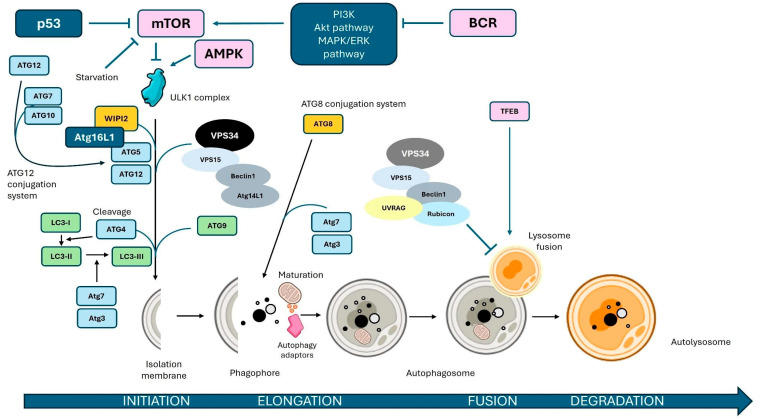
Autophagy pathway.

This intricate autophagic process showcases its significance in cellular regulation, with each stage offering potential therapeutic intervention points in cancer treatment. As a multifaceted process, autophagy integrates several signaling pathways, underscoring its role in cellular adaptation and survival under stress (Figure 1).

### 2.2. Types of Autophagy

Autophagy encompasses two main mechanisms: microautophagy and macroautophagy. Whereas microautophagy is a non-selective process where cellular components are directly engulfed through membrane invaginations of the lysosome or vacuole, macroautophagy (commonly referred to as autophagy) involves recycling damaged or dysfunctional organelles within an autophagosome that later fuses with the lysosome to degrade its contents [31,35]. On the other hand, autophagy can be classified as selective or non-selective. Whereas non-selective autophagy degrades cellular materials without prior recognition, primarily maintaining basic cellular functions, selective autophagy is a specific chaperone-mediated process that targets harmful cellular elements such as damaged proteins, toxic aggregates, or invasive pathogens for lysosomal degradation [36,37].

## 3. Autophagy Regulatory Drugs

In recent years, there has been a significant focus on drugs targeting the autophagy pathway, largely due to the role of autophagy in cellular homeostasis, cancer, and other diseases. Autophagy modulators are classified broadly as autophagy inducers and autophagy inhibitors, each with different mechanisms and clinical potential.

### 3.1. Autophagy Inducers

Autophagy inducers have shown promise in cancer and neurodegenerative diseases, where promoting the clearance of damaged cellular components can be beneficial. Key drugs include rapamycin, resveratrol, and spermidine.

- Rapamycin: Rapamycin is a well-characterized mTORC1 inhibitor that blocks the mTOR signaling pathway, a central regulator of autophagy. By inhibiting mTORC1, rapamycin induces autophagy initiation and has demonstrated efficacy in promoting autophagic cell death in cancer cells, particularly in those resistant to apoptosis [38,39,40]. Beyond its anticancer properties, rapamycin has shown therapeutic potential in other age-related diseases, emphasizing its broader clinical applications [41].
  Limitations: Rapamycin’s inhibition of mTORC1 can lead to side effects, including suppression of T-cell proliferation [42,43], which is critical for immune responses against pathogens. Additionally, Rapamycin’s inhibition produces thrombocytopenia, hyperlipidemia, insulin resistance and hyperglycemia [42,43,44,45]. These facts limit its long-term use and application as a preventive tool. Strategies to overcome these side effects include intermittent dosing, selective inhibition of mTORC1 (without affecting mTORC2) and combination therapies with metformin [45,46].
  Resveratrol: Resveratrol is a natural polyphenol found in plants, including knotweed and berries. Chemically, it is a stilbene derivative composed of two phenyl rings connected by an ethylene bridge. This unique chemical structure underpins its biological activities, particularly its ability to scavenge ROS and regulate signaling pathways involved in cellular stress responses [46,47]. Resveratrol, commonly found in dietary sources such as grapes and red wine, has been shown to activate autophagy through the inhibition of the mTOR pathway and the activation of AMPK, which further suppresses mTOR signaling [48]. Additionally, Resveratrol exhibits significant anti-tumor properties by inducing both apoptosis and autophagy, especially in cancers characterized by high oxidative stress [49].
  Limitations: Resveratrol may impair glucose metabolism, potentially leading to insulin resistance and hyperglycemia. As Rapamycin, it may suppress immune responses by affecting T-cell proliferation and cytokine production, increasing susceptibility to infections or impairing wound healing [50]. Prolonged inhibition of mTORC1 has also been linked to liver inflammation and gastrointestinal discomfort or diarrhea, although these effects are generally mild [51]. Mitigation strategies to minimize side effects include intermittent dosing, targeted delivery (such as CNS-specific formulations), and combination therapies with agents that counteract metabolic dysregulation (e.g., metformin) [52].
- Spermidine: Spermidine is an endogenous polyamine that enhances autophagy by inhibiting acetyltransferases and promoting the deacetylation of ATG proteins, which are essential for autophagy initiation. It has demonstrated anti-aging, anticancer, and geroprotective effects, reducing oxidative stress and lowering the incidence of cardiovascular and neurodegenerative diseases [53,54,55]. Studies show that spermidine levels increase during fasting or caloric restriction across species, and blocking its synthesis impairs fasting-induced autophagy and negates the lifespan- and healthspan-extending effects of these interventions. Spermidine mediates these effects through autophagy induction and hypusination of the translation regulator eIF5A, positioning the polyamine–hypusination axis as a conserved metabolic hub for longevity and health benefits [56,57].
  Limitations: Spermidine’s interaction with mTORC1 can disrupt nutrient sensing and energy metabolism, potentially leading to imbalances in amino acid homeostasis and energy utilization, particularly during nutrient deprivation [58]. In some contexts, spermidine may also impair immune cell proliferation and function, increasing susceptibility to infections or reducing the body’s ability to respond to stressors. While beneficial in cancer therapy [59,60], spermidine-induced inhibition of mTORC1 can suppress normal cell proliferation and growth, potentially affecting healthy tissues. Moreover, spermidine has been shown to enhance apoptosis via mitochondrial pathways, which is advantageous for targeting cancer cells but could harm normal cells if not carefully controlled. Given that excessive polyamine accumulation has been linked to genomic instability and neurodegeneration due to deregulated protein degradation and nutrient sensing pathways, dosing and long-term use of spermidine need to be considered carefully. Mitigation strategies to reduce spermidine-associated risks include controlled dosing, tissue-specific delivery systems, and combination therapies with agents that counteract its adverse effects (e.g., metabolic regulators).

### 3.2. Autophagy Inhibitors

Even though autophagy supports cell survival under stress conditions, excessive autophagy can lead to autophagic cell death. Inhibitors of autophagy are, therefore, relevant in treating cancers that exploit autophagy for survival.

Chloroquine (CQ) and Hydroxychloroquine (HCQ): Both chloroquine and hydroxychloroquine are lysosomotropic agents that disrupt lysosomal acidification, thereby inhibiting the fusion of autophagosomes with lysosomes [61]. By blocking the final stages of autophagy, these agents induce cell death in cancer cells that rely on autophagy for survival. CQ and HCQ have been extensively studied in clinical trials and have demonstrated significant potential, particularly when used in combination with other cancer therapies, highlighting their promise as adjunctive treatments in oncology [62,63,64].

Bafilomycin A1: An inhibitor of vacuolar H+-ATPase, Bafilomycin A1 prevents lysosomal acidification in a manner like CQ [65]. It has shown significant anti-tumor properties, particularly in cancers that are highly dependent on autophagy [66,67]. Due to its ability to effectively block autophagic flux, Bafilomycin A1 has become an invaluable tool in research for studying the autophagy pathway and its implications in cancer biology.

3-Methyladenine (3-MA): 3-MA is a well-established inhibitor of autophagy that acts by blocking class III phosphoinositide 3-kinase (PI3K), a key regulator in the early stages of autophagosome formation. By inhibiting this kinase, 3-MA prevents the initiation of autophagy, thereby reducing the formation of autophagosomes [68]. Although its use in clinical applications is limited due to potential off-target effects and the incomplete inhibition of autophagy, 3-MA remains an indispensable tool in experimental research [69]. It is widely used to investigate autophagy’s roles in cellular processes like survival, stress response, and disease progression, enabling researchers to explore its contribution to homeostasis and its involvement in diseases such as cancer, neurodegeneration, and infection [70,71,72]. 

Therefore, modulating autophagy in cancer therapy requires a deep understanding of the cellular and molecular context of each tumor. Developing effective strategies will depend on how we predict and manipulate the balance between the cytoprotective and cytotoxic effects of autophagy in cancer cells.

## 4. Oxidative Stress and ROS

Cell metabolism involves anabolic and catabolic pathways that maintain energy balance. In multicellular organisms, oxygen is the main substrate for aerobic respiration, producing adenosine triphosphate (ATP) [73,74]. Under physiological and resting conditions, most of the oxygen consumed by cells is reduced to water via cytochrome oxidase activity, but 1–2% generate ROS through electron transfer events or reduction/oxidation (redox) reactions within the mitochondria [75].

ROS were first identified as free radicals in skeletal muscle with harmful effects on cells [76]. These specific oxygen-containing molecules are highly reactive and unstable, classified into non-radical and free radicals based on unpaired electrons (Table 1) [77]. Recent research highlights their dual nature [78,79]. While they function as pleiotropic physiological molecules at the baseline cell homeostatic state, involved in signaling pathways, immune defense and cell differentiation, elevated ROS levels cause cellular damage and contribute to disease development [77,80].

Cells maintain ROS homeostasis by tightly regulated biological mechanisms through a balance between ROS production and scavenging mechanisms. Disruption of this balance, typically due to elevated ROS levels, results in oxidative stress, a state characterized by the disturbance of cellular redox homeostasis [81,82]. Understanding the complex interplay between ROS, oxidative stress, antioxidants and cellular metabolism is crucial for developing targeted interventions in several diseases associated with redox imbalance such as cancer [83,84].

### 4.1. Sources of ROS in Cells

ROS are well-known to be produced from two primary sources: endogenous and exogenous factors like radiation, pollutants, cigarette smoke, and nutrition [85]. Endogenously, ROS are mainly produced by mitochondria and nicotinamide adenine dinucleotide phosphate (NADPH) oxidases (NOXs), with other enzymes such as endoplasmic reticulum (ER) oxidases, peroxisomes, superoxide dismutases (SODs), xanthine oxidoreductase, nitric oxide synthases (NOSs), lipoxygenases, prostaglandin synthases, cyclooxygenases and cytochrome P450 also contributing to their production [80,86,87].

The electron transport chain (ETC) in mitochondria is crucial for ATP production through oxidative phosphorylation. Electrons from metabolic substrates are transferred through protein complexes in the inner mitochondrial membrane (complexes I-IV), reducing oxygen to water and generating a proton gradient that drives ATP synthesis via the ATP synthase (complex V) [88]. Normally, ROS production in the ETC is low and regulated, serving as signaling messengers for processes like metabolism, apoptosis, and stress adaptation. However, complexes I and III are major ROS sources, leaking electrons that generate superoxide anion radical (O_2_^−^) and hydrogen peroxide (H_2_O_2_) [89,90,91]. When ETC function is impaired, this electron leak can overwhelm antioxidant defenses, causing mitochondrial dysfunction, macromolecular damage, and disrupting signaling pathways linked to disease, or triggering immune responses as danger-associated molecular patterns (DAMPs) [92,93].

On the other hand, NOXs are a family of enzymes in the plasma membrane that play a key role in cytoplasmic ROS production. In humans, the NOX family includes seven members (NOX1-NOX5, DUOX1 and DUOX2), which primarily produce ROS, unlike other cellular sources where ROS are by-products [94]. NOXs catalyze electron transfer from NADPH to oxygen, generating O_2_^−^ that can form other ROS like H_2_O_2_ or hydroxyl radicals (-OH). NOX-derived ROS are involved in host defense (e.g., NOS2 in phagocytes killing pathogens) [95], signaling pathways (e.g., NOX4 regulating vascular tone [96], and thyroid hormone biosynthesis (DUOX-produced H_2_O_2_) [97]. Dysregulation of NOXs leads to excessive ROS, contributing to oxidative stress and damage in various diseases, including cardiovascular, chronic inflammatory diseases, blood disorders, and tumors [98].

The ER and peroxisomes are key cellular compartments for ROS production. In the ER, H_2_O_2_ is generated during oxidative protein folding by enzymes like protein disulfide isomerase and oxidoreductin-1. In peroxisomes, H_2_O_2_ is produced during fatty acid β-oxidation, amino acid catabolism, and purine metabolism, and can be neutralized by CATs. However, excessive ROS levels can overwhelm antioxidant defenses, contributing to lipid peroxidation and oxidative stress [99,100].

Last, exogenous ROS from sources like radiation, pollutants, toxins, or therapeutic drugs can amplify endogenous ROS production by damaging organelles and activating ROS-producing enzymes like NOXs [101]. These ROS regulate signaling pathways but can cause oxidative damage to DNA, lipids, and proteins when levels exceed antioxidant defenses [102]. This interaction contributes to diseases such as hematological malignancies, solid tumors, and neurodegenerative and cardiovascular disorders, emphasizing the importance of maintaining redox balance to prevent pathological outcomes [103].

### 4.2. Antioxidant Defense Systems

Cells maintain redox homeostasis by balancing ROS production and antioxidant defenses. ROS production is initially limited by mitochondria during OXPHOS, with NOXs and other oxidases suppressed in a preventive phase. When ROS levels rise, cells activate antioxidant systems to neutralize potential damage [81]. Key defense mechanisms, including autophagy, counteract ROS through a dynamic interplay of oxidants, antioxidants, and cellular responses [104,105].

Antioxidant defense components are categorized based on their synthesis, nature, or function and can be divided into first-line and second-line defenses. The system is regulated by cellular mechanisms to cope with chronic oxidative stress [106]. Enzymatic components like SODs, catalases (CATs) and glutathione peroxidases (GPx) provide rapid first-line defense by neutralizing ROS. Non-enzymatic components like reduced glutathione (GSH), and peroxiredoxin/thioredoxin (TRX) system also contribute to defense. Second-line defenses include ubiquinol (Coenzyme Q10) and exogenous antioxidants, such as vitamins, minerals, flavonoids, and carotenoids, which further support ROS neutralization [107] (Table 2).

Finally, despite preventive and detoxification measures, oxidative damage can occur due to prolonged exposure to ROS. Hence, cells have evolved an adaptive third-line defense response to restore redox balance by upregulating antioxidant defenses, repairing oxidatively damaged molecules, and removing defective cellular components. Key mechanisms of this cellular response include: (1) activation of antioxidant enzymes; (2) nuclear factor erythroid 2-related factor2 (Nrf2); (3) autophagy; (4) mitophagy; (5) non-enzymatic defense; and (6) metabolic reprogramming. For instance, stress signals dissociate Nrf2 from its inhibitory complex (Kelch-like ECH-associated protein 1, Keap1), allowing its translocation to the nucleus and activation of target genes encoding antioxidant enzymes such as SODs, CATs or GPxs, while also promoting the catabolic process to degrade damaged organelles, misfolded proteins and other toxic aggregates by inducing expression of ATGs (e.g., p62/SQSTM1). Furthermore, impaired autophagy leads to p62 accumulation, which competes with Nrf2 for binding KEAP1, enhancing Nrf2 activation [108]. This cross-regulation is critical for protecting cells from oxidative stress, and emerging evidence highlights the potential of targeting the Nrf2–autophagy axis in therapeutic interventions for neurodegenerative diseases, cardiovascular disorders, and cancer [11,109,110,111].

### 4.3. Redox Signaling

**Figure 2:**
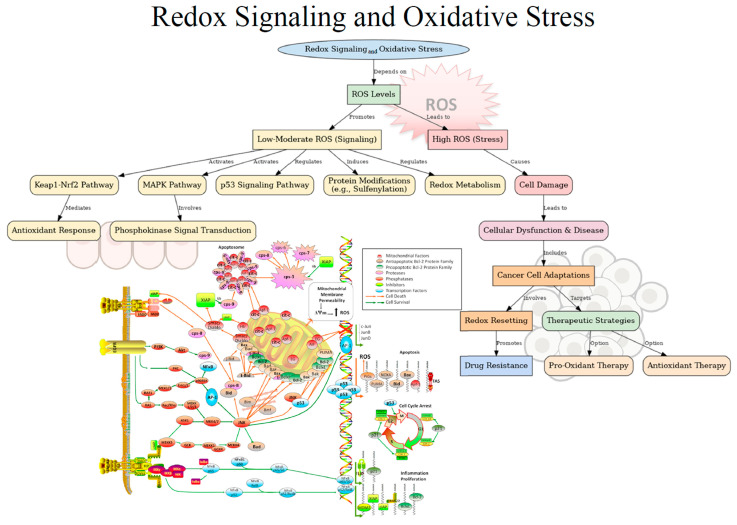
Redox signaling and oxidative stress.

Redox signaling and oxidative stress are closely related but distinct, with ROS playing a key role in determining cellular fate depending on their concentration and context (Figure 2). Redox signaling involves low to moderate ROS levels that regulate biological processes, while oxidative stress occurs when ROS levels surpass the cell’s antioxidant capacity, leading to cellular damage, disrupted signaling, and disease [112].

Spatial and temporal regulation is central to redox signaling. ROS are transiently produced to enable dynamic responses, localized to specific cellular compartments such as mitochondria, the ER, or the plasma membrane, ensuring precise outcomes. Key targets are redox-sensitive residues, particularly cysteines and methionines, whose reversible modifications regulate protein activity [112,113]. ROS like O_2_^−^, H_2_O_2_, and OH^−^ are essential for oxidative stress and activating signaling cascades [80]. These signals are propagated through complex communication networks, influencing processes like mitogen-activated protein kinase (MAPK) cascades and p53 signaling, leading to responses such as cell cycle arrest, senescence, or apoptosis [114]. Thus, redox signaling plays a key role in regulating cellular processes, including antioxidant response, phosphokinase signal transduction and redox metabolism [114,115]. Understanding redox chemistry involves considering reaction kinetics. Redox signaling requires an oxidant (electrophile) reacting with a reductant (nucleophile). Unlike conventional second messengers like cyclic adenosine monophosphate (cAMP), redox signaling uses molecules with greater potential for non-specific reactions. There are two main types of redox reactions: oxidation, where the oxidant accepts electrons, and leaving the reducing agent in a more oxidized state. The oxidant may take one electron (a free radical reaction) or two, leading to the oxidation of target proteins. Two-electron oxidations predominate in redox signaling, as free radicals are more likely to trigger further reactions. These reactions can alter protein function, gene expression, or post-translational modifications [115,116]. The second type involves the formation of a covalent bond between the reductant and oxidant, where atoms share electrons instead of fully losing or gaining them [115]. However, as it will be discussed in more detail in subsequent sections, dysregulated redox reactions and ROS production play crucial roles in blood and solid cancers, where ROS act as second messengers regulating cell proliferation, death, and chemoresistance [117,118,119].

Upon therapy, some tumor cells undergo redox resetting, acquiring a new balance with higher ROS levels and stronger antioxidant systems. This enables cancer cells to resist anticancer drugs through mechanisms like increased drug efflux, altered drug metabolism, mutated targets, activated pro-survival pathways, and reduced cell death [115]. Understanding these mechanisms offers promising strategies to overcome drug resistance and improve treatment outcomes [120]. Furthermore, during metastasis, cancer cells face significant oxidative stress as they migrate through diverse environments. To survive, they undergo reversible metabolic changes that enhance oxidative stress resistance. However, oxidative stress can also induce ferroptosis, limiting the survival of metastasizing cells [112,121]. Given the role of ROS in cancer, therapeutic strategies often target redox status. Pro-oxidant therapies aim to increase oxidative stress in cancer cells, while antioxidant therapies seek to reduce it. Natural substances from plants have chemopreventive potential by modifying redox status [122,123,124]. Therefore, redox reactions and oxidative stress are central to cancer biology, affecting tumor development, progression, and response to treatment.

## 5. Crosstalk Between Autophagy and Oxidative Stress

**Figure 3:**
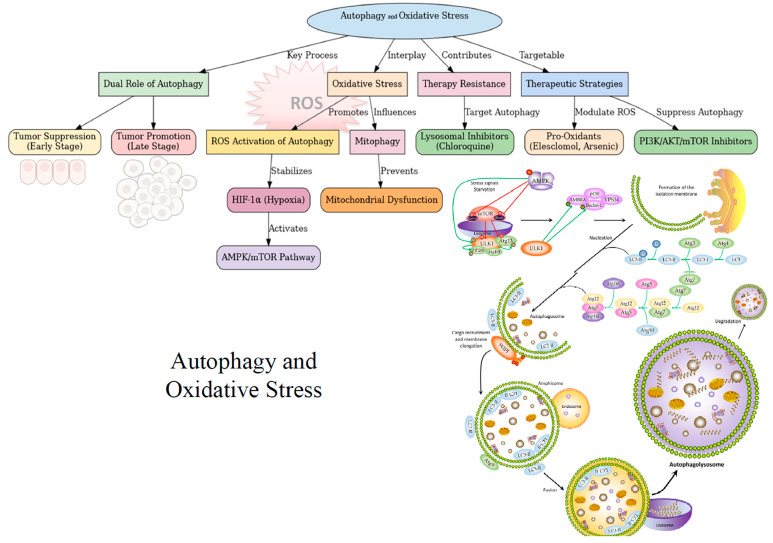
Autophagy and oxidative stress in hematological malignancies.

Autophagy and oxidative stress are tightly regulated processes significantly influence cancer onset and tumor progression [125,126,127] (Figure 3). Their interplay can suppress or promote tumor growth, depending on the context [128]. In early tumorigenesis, autophagy acts as a tumor suppressor mechanism by degrading oncogenic molecules, damaged organelles, and misfolded or polyubiquitinated proteins [129,130]. It also helps reduce oxidative stress and cytoplasmic debris [131,132,133], which have been related to genomic instability and the accumulation of oncogenic mutations [129,130,134]. However, in later stages with established tumors and during cancer progression, autophagy influences tumor metabolism, supporting cell survival by sustaining energy needs for DNA repair, adapting to the microenvironment [135], and modulating ROS production, metabolic reprogramming, immune evasion, metastasis, and resistance to treatments [136].

### 5.1. ROS as Inducers of Autophagy

Multiple studies have suggested that the crosstalk between autophagy and oxidative stress is mediated by redox-sensitive proteins, which contain specific amino acid residues particularly susceptible to oxidation or reduction [137,138]. These proteins modulate the intracellular redox environment, often shifting it towards a more oxidizing state [139]. During starvation, exposure to H_2_O_2_ triggers the efficient extrusion of GSH via the MRP1 drug efflux pump. This process activates AMPK through S-glutathionylation of specific reactive cysteine residues (Cys299 and Cys304) in α and β subunits, which phosphorylates and activates key autophagy proteins, including ULK1 [140], mTORC1, and PIK3C3/VPS34 [141,142,143,144]. Since S-glutathionylation can induce autophagy without other stimuli, thiol redox homeostasis appears crucial for regulating autophagy. In addition to AMPK, several proteins involved in the autophagy initiation, such as the ubiquitin-like systems ATG7-ATG3 [143,145] and ATG7-ATG10 [143], Beclin-1 [140], PI3K [140], members of Rab GTPase [146,147], PTEN (Cys124 and Cys71) [148] and SQSTM1/p62 [140], are modulated by oxidation of specific cysteine residues. In support of the hypothesis suggesting the regulation of autophagy-related proteins through the oxidation of cysteine residues, it has previously been demonstrated that the H_2_O_2_-mediated oxidation of cysteine residues in ATG4, ATG3, and ATG7 proteins is essential for inhibiting their hydrolyzing activity on LC3-II, facilitating proper autophagosome elongation [143,144]. H_2_O_2_ inactivates ATG4B by oxidizing Cys81 near the catalytic site [143,149] and reduces the interaction between ATG3 and ATG7 with LC3, preventing LC3 lipidation and autophagy initiation [143]. In addition, mutations affecting Cys292 and Cys361 residues in the *ATG4B* locus have been linked to increased autophagy flux, likely by altering the protein’s redox sensitivity [150,151]. Furthermore, under oxidative stress, AKT forms disulfide bonds between Cys297 and Cys311, leading to its dephosphorylation and inactivation, which reduces mTORC1 activity and induces autophagy [152]. Similarly, ROS increase AMPK phosphorylation and activity, thus leading to the induction of autophagy through the inhibition of mTORC1 activity and PI3K-AKT signaling [153]. Furthermore, ROS oxidize ATM, promoting the formation of intramolecular disulfide bonds at Cys-2991. This oxidation activates ATM independently of the DNA damage response pathway and induces TSC2-mTOR signaling pathway, thereby promoting autophagy initiation [154]. ROS can also activate p53, which induces the transcription of sestrin proteins that promote autophagy initiation through AMPK activation and mTORC1 inhibition via the assembly of TSC1 and TSC2 [153]. Finally, it has been reported that H_2_O_2_ induces the translocation of TFEB from the cytoplasm to the nucleus. This translocation triggers autophagy and lysosomal biogenesis as a defensive response against oxidative damage [155]. Although the translocation mechanism is not fully understood, it is believed to be directly induced by ROS, which oxidize specific cysteine residues in TFEB, TFE, and MITF, leading to enhanced expression of genes implicated in the autophagy-lysosome pathway [156], thus linking redox signaling with autophagic regulation.

In addition to the post-transcriptional regulation of autophagy by oxidative stress, it has been reported the existence of redox-independent relationship between autophagy and antioxidant response, primarily mediated by the p62/Keap1/Nrf2 pathway [131,140]. Furthermore, it has been shown to selectively target oxidized and damaged biomaterials for lysosomal degradation [157], reducing oxidative stress and promoting cell survival.

### 5.2. Autophagy as Regulator of Oxidative Stress

Autophagy regulates oxidative stress by clearing damaged organelles [137], oxidized proteins [158], and protein aggregates [159], as well as by reducing ROS levels through different pathways, including the regulation of TFEB [156]. It is well known that autophagy plays a key role in maintaining cellular homeostasis by selectively targeting specific organelles for degradation, including mitochondria (mitophagy), peroxisomes (peroxiphagy), the ER (reticulophagy) and lysosomes (lysophagy) [132,137]. Once formed, the autophagosome can engulf these organelles or harmful protein aggregates, which are then degraded by lysosomal enzymes [160]. This process is vital for preventing ROS accumulation particularly from dysfunctional mitochondria [161,162], peroxisomes [143,163] and lysosomes [132,156]. It also helps maintain the balance between ROS production and scavenging [164,165], facilitating the recycling of their components for energy production and biosynthesis [160,166]. ROS from mitochondria are mainly involved in oxidative phosphorylation reactions within the inner mitochondrial membrane. These ROS are regulated by classical scavengers, including SOD family proteins and the GSH redox system, which sequentially convert O_2_^−^ into H_2_O_2_, and then reduce it to O_2_ and H_2_O [167]. When mitochondria are dysfunctional, ROS accumulate leading to cellular damage [168] and activating autophagy. Conformational changes in the mitochondrial membrane trigger autophagy activation through Parkin-dependent ubiquitination [169] and the BNIP3-NIX-FUNDC1 mitochondrial adaptor pathways. When Parkin is phosphorylated by PTEN-induced putative kinase 1 (PINK1), it ubiquitinates outer membrane proteins (VDAC1, Mfn1 and Mfn2) [158,170], as well as other proteins such as fission protein (FIS) and its adaptor (TBC1D15), and mitochondrial translocases (TOMM20 and TOMM70) [170]. Once ubiquitinated and tagged for proteasomal degradation, these proteins bind to autophagy cargo receptors (SQSTM1, NDP52 and optineurin) [158], promoting mitochondrial engulfment by the autophagosome. This process is also activated by Rab signaling proteins, including RABGEF1, RAB5 and RAB7A [171,172], and autophagy receptors such as p62, TAX1BP1, and CALCOCO2 [170]. Furthermore, the BNIP3-NIX-FUNDC1 mitochondrial adaptor pathway promotes mitochondrial attachment to the autophagosome. This step is positively controlled by ULK1 and Src [173,174] and involves the recruitment of WIPI proteins (WIPI1, WIPI2 and WIPI3) to facilitate the recruitment of downstream proteins of the autophagy machinery [175].

On the other hand, the engulfment of peroxisomes plays a key role in modulating oxidative stress. These organelles are involved in lipid metabolism, ketogenesis, and the metabolism of cholesterol and isoprenoids [176]. They contain acyl-CoA (ACOX) and D-amino oxidases, which generate intracellular H_2_O_2_ [177], as well as xanthine oxidases and small ETCs in their membranes that produce anion superoxide (O_2_^−^) [178]. When peroxisomes are defective or damaged, they cause an elevation in intracellular ROS levels, activating ATM through the oxidation of specific cysteine residues, including Cys2991. This oxidation results in the formation of multiple intracellular disulfide bonds, promoting ATM dimerization [179]. Once activated, ATM promotes AMPK and ULK1 activation while inhibiting mTORC1 to induce autophagy. Additionally, ATM phosphorylates PEX5 at Ser141 and promotes its mono-ubiquitination at K209 [180], facilitating its recognition by p62 and NRB1. These adaptor proteins, in association with LC3, guide the autophagosome to the damaged peroxisomes [181]. Another peroxisomal protein recognized by p62 and NRB1 after undergoing oxidative modifications is PEX14, which is implicated in the timely removal of dysfunctional peroxisomes. H_2_O_2_-induced phosphorylation of PEX14 at Ser232 inhibits the peroxisomal import of CAT in vivo and disrupts the interaction of CAT with the PEX14-PEX5 complex in vitro [182].

Regarding reticulophagy and lysophagy, there is solid evidence suggesting that they help in eliminating damaged ER and lysosomes. Reticulophagy is activated during ER stress and helps in maintaining ER homeostasis by degrading damaged ER components, which can be triggered by oxidative stress through the unfolded protein response (UPR) [183]. Additionally, reticulophagy indirectly influences oxidative stress by preserving mitochondrial function, as intact mitochondria are observed during excessive ER-phagy [184]. Reticulophagy helps in reducing ER stress-induced ROS production, which can otherwise damage mitochondria [185]. In addition, ER stress activates the Nrf2 transcription factor, which enhances the expression of antioxidant response genes, thus protecting mitochondria from oxidative damage [185]. One key mechanism involves the PERK pathway, where the ER stress sensor PERK phosphorylates and activates NRF2, causing it to dissociate from its repressor KEAP1 and translocate to the nucleus [186,187]. Recent studies have demonstrated that PERK activation stimulates NRF2 expression via the transcription factor ATF4, suggesting that NRF2 has a central role in preventing oxidative damage [186]. A noncanonical pathway also involves the autophagy receptor p62/SQSTM1, which binds and degrades KEAP1, thus facilitating NRF2 activation. Once activated, NRF2 induces the expression of antioxidant genes such as *NQO1* and *HMOX1/HO-1*, which help in neutralizing ROS and protect mitochondria from oxidative damage [188]. Furthermore, NRF2 activation promotes components of the UPR, including XBP1 and ATF6α, contributing to ER integrity and protein homeostasis [189,190]. By coordinating these protective responses, NRF2 activation helps maintain redox balance, reduce mitochondrial oxidative damage, and support cell survival during stress [188]. Besides these mechanisms, reticulophagy supports mitochondrial quality control by maintaining ER function, which is essential for protein folding [191,192,193,194], lipid synthesis [195], and mitochondrial membrane integrity [196,197]. It also regulates mitophagy by providing membrane sources for autophagosome formation and influences mitochondrial energy metabolism by regulating lipid homeostasis [198] and calcium signaling between the ER and mitochondria [197,199], affecting ATP production. Likewise, reticulophagy impacts mitochondrial dynamics, including fission and fusion [185], by modulating the structure of the ER, which is essential for mitochondrial network formation and cellular stress adaptation. Reticulophagy also controls oxidative stress by influencing lipid metabolism [200] and lipid droplets (LDs) homeostasis, which store excess lipids. LDs play a protective role by preventing lipotoxicity and the toxic effects of unesterified lipids [201,202,203]. Conversely, changes such as the accumulation of free fatty acids, cholesterol and ceramide lead to lysosomal membrane permeabilization and lipid-ROS production [184,198]. The ER and mitochondria are connected through structures known as mitochondria-associated membranes [204,205,206], which are crucial for lipid synthesis and exchange [207,208], particularly involving phospholipids like phosphatidylcholine, phosphatidylethanolamine, diacylglycerol, and cholesterol [209]. Disruptions in lipid metabolism at these sites can destabilize lysosomal membranes, leading to lipotoxicity [210,211], accumulation of lipid hydroperoxides, and increased membrane permeability [212,213]. This destabilization facilitates the release of ROS and damaging contents from ribosomes and lysosomes, such as cathepsins, exacerbating oxidative stress [214].

Similarly to reticulophagy, ribophagy, and lysophagy, the selective autophagy of damaged ribosomes and lysosomes can mitigate oxidative stress [215]. Given that these processes are faster compared than the autophagy of entire organelles, it has been proposed that they are selective mechanisms [200,216,217,218]. Among these specific autophagic processes, lysophagy has gained significant attention as it supports mitochondrial quality control by maintaining lysosomal function, which is critical for mitophagy, and reducing ROS production [212,219]. Recent studies have suggested that ubiquitination plays a key role in the regulation of both lysophagy and ribophagy [216,220]. Ubiquitin-based modifications are commonly involved in the selective elimination of cellular structures, suggesting they could dictate which ribosomal and lysosomal components are targeted for degradation. Upon lysosomal damage, extensive ubiquitination of lysosomal proteins occurs [221], involving both K63-linked and K48-linked ubiquitin chains that serve as recruitment platforms for autophagy receptors, enabling the identification of damaged organelles [222,223]. This ubiquitination cascade relies on E1 ubiquitin-activating enzymes, E2 ubiquitin-conjugating enzymes like UBE2QL1, and E3 ubiquitin ligases such as TRIM16 and SCF FBXO27 [222]. UBE2QL1 is particularly critical, as its depletion reduces ubiquitination and disrupts lysophagy [224]. Temporal dynamic studies have revealed that K63 chain formation occurs within 30–60 min of damage, while K48 chains peak after 2–3 h [222]. These ubiquitin chains recruit autophagy receptors, such as p62/SQSTM1, TAX1BP1, and Optineurin, which link damaged lysosomes to the autophagy machinery [225]. Additionally, ubiquitination facilitates the recruitment of the AAA-ATPase VCP/p97 and the TRIM16-Galectin-3 complex, which, along with factors like ATG16L1 and ULK1, drive local phagophore formation [222,223]. This orchestrated process ensures the efficient removal of damaged lysosomes, maintaining cellular homeostasis.

In ribophagy, the Ubp3-Bre5 complex interacts and controls the ubiquitination of ATG19 [226], a receptor in the cytoplasm-to-vacuole targeting pathway [227]. ATG5-ATG19 autophagy interaction motifs (AIM) interaction competes with the ATG8-ATG19 AIM interaction [228], suggesting a regulatory mechanism involving ubiquitination and deubiquitination activities. In addition, it is also supported by the fact that decreased levels of the ubiquitin ligase Rsp5, along with the deletion of Ubp3, impair ribosome turnover, although other cytoplasmic proteins are still degraded by autophagy [229]. These findings underscore the importance of both ubiquitination and deubiquitination in regulating ribophagy and suggest the importance of understanding the precise mechanisms behind these processes and its regulation.

Finally, it is important to highlight the role of lipophagy in cellular lipid metabolism and homeostasis, particularly under oxidative stress conditions [230]. High glucose levels, for example, can activate lipophagy to alleviate lipid accumulation by promoting LD breakdown and enhancing mitochondrial β-oxidation, with oxidative and ER stress pathways acting as key regulators [231]. ROS also triggers lipophagy through the activation of transcription factors like TFEB via lysosomal calcium release, creating a feedback mechanism to mitigate oxidative damage [232]. However, prolonged oxidative stress can impair lipophagy [183,209], as seen in endothelial cells exposed to oxidized low-density lipoprotein (ox-LDL), where reduced lipophagic activity leads to lipid accumulation and cellular damage [233]. Interestingly, exposure to ox-LDL also induces oxidative stress in liver, increasing LD enriched with cholesteryl ester hydroperoxidases and deregulating genes like *SREBP1*, *FASN* and *DGAT1* [234]. These findings underscore the critical role of lipophagy in lipid homeostasis [231] and its dysfunction under oxidative stress, contributing to diseases like atherosclerosis and non-alcoholic fatty liver disease (NAFLD) [230,234]. Furthermore, lysosomes are central to this process, as impaired lysosomal function exacerbates oxidative stress and metabolic dysfunctions, highlighting the interplay between lysosomal activity, ROS production, and lipophagy in cellular health.

Although the information above links mitophagy, reticulophagy, ribophagy, lysophagy, lipophagy and oxidative stress, further research is needed to fully understand their roles in maintaining cellular redox balance and their contributions to diseases such as NAFLD, neurodegenerative diseases, and cancer.

## 6. The Role of Autophagy and Oxidative Stress in Hematological Malignancies

Autophagy and oxidative stress play crucial roles in the development, progression, and treatment of hematological malignancies. Disruption in autophagy and oxidative imbalance during hematopoiesis can lead to malignant transformation and increased cell proliferation [21] and it may have different biological effects depending on the specific tumor type, genetic context, and stage of development [235]. The complex interplay between autophagy and oxidative stress significantly impacts malignant cell survival, drug resistance, and therapeutic outcomes.

### 6.1. Autophagy and ROS as Modulators of Tumor Survival, Treatment and Disease Progression

In the early stages of hematological cancers, autophagy serves as a survival mechanism that helps cancer cells cope with metabolic stress caused by rapid proliferation, nutrient deprivation, or hypoxia. This adaptive response allows tumor cells to maintain viability under harsh conditions and contributes to their persistence and potential relapse. Autophagy enables cancer cells to recycle intracellular components, providing energy and essential building blocks during metabolic stress. This process is particularly important in apoptosis-defective cells, allowing them to survive prolonged nutrient and oxygen deprivation [59,236]. In leukemic cells, hypoxia-induced autophagy has been shown to support the survival of leukemia stem cells (LSCs), contributing to chemoresistance and disease progression [237]. Additionally, autophagy facilitates tumor dormancy by enabling residual cancer cells to endure metabolic deprivation. These dormant cells can later re-enter the cell cycle and drive relapse when conditions become favorable [238,239]. The ability of autophagy to maintain cellular homeostasis under stress resembles a “hibernation-like” state that enhances long-term survival [236,238]. While autophagy supports cancer cell survival, its suppression in early tumor stages can lead to increased genomic instability, inflammation, and necrotic cell death, promoting tumorigenesis [236,237].

On the other hand, in advanced stages, autophagy becomes a pro-survival mechanism that sustains malignant growth and resistance to therapy [236,240]. Therapeutically, inhibiting autophagy may improve treatment outcomes in hematological cancers by sensitizing cancer cells to stress-induced death, particularly in apoptosis-defective tumors [236,240]. However, the paradoxical role of autophagy as both a tumor suppressor and promoter complicates therapeutic strategies. Careful timing and context-specific targeting are critical to avoid unintended consequences. Clinical trials are currently investigating the efficacy of therapies targeting autophagy and oxidative stress in combination with traditional treatments to enhance patient outcomes. Strategies aimed at oxidative stress focus on two key approaches: mitigating ROS-induced damage to healthy tissues or leveraging elevated ROS levels to selectively target cancer cells. Increasing evidence highlights the potential of combination therapies that concurrently modulate autophagy and oxidative stress, offering a promising avenue for more effective cancer treatments. For example, combining autophagy inhibitors with pro-oxidants has shown synergistic effects in preclinical models, as the inhibition of autophagy sensitizes cancer cells to ROS-induced death [18]. Additionally, targeting upstream regulators of autophagy and oxidative stress, such as the PI3K/AKT/mTOR axis, offers a promising avenue for integrated therapeutic strategies.

### 6.2. ROS and Autophagy in Hematological Malignancies

#### 6.2.1. Leukemias

##### Chronic Lymphocytic Leukemia

In CLL, oxidative stress levels are higher compared with normal B cells. The main source of ROS in CLL cells is mitochondria, which also has an increased mitochondrial mass. Mitochondrial ROS, specifically superoxide and hydrogen peroxide, are products of mitochondrial respiration and play a role in B-cell receptor (BCR) signaling by modulating cellular metabolism. This process involves oxidative phosphorylation and highlights the differences between naïve B cells and anergic B cells [241]. Higher levels of ROS produce genomic instability and DNA damage which affects disease progression. Mitochondrial DNA mutations (mtDNA) can increase the nitric oxide (NO) levels, which have an influence on mitochondrial biogenesis [242]. Inhibition of NOS, the enzymes that produce NO and oxidative stress, can modify this process. It is demonstrated that L-NAME, an inhibitor of NOS, induces apoptosis in CLL cells by the reduction in the NO production affecting the oxidative stress pathways and the mitochondrial biogenesis [242,243].

Additionally, PI3K/AKT signaling pathways play a key role for cell proliferation and survival. It is overexpressed in CLL cells due to the inhibition of the SH1P phosphatase, which usually inhibits this pathway. Restoring the SHIP1 activity could be a potential target for CLL by limiting this pathway and promoting cell death [244]. Higher levels of phosphorylated STAT3 in Ser727 (pSTAT3Ser727) in mitochondria is another significant mechanism for the CLL. Overexpression of STAT3 improves the antioxidant defenses of the CLL cells, improving their survival. So, it could be a potential target therapy to reduce malignant B cells in CLL [245]. In conclusion, these processes highlight the role of the mitochondrial function in the PI3K/AKT signalization and the antioxidant defense mechanism in CLL, suggesting several therapeutics targets in future treatments.

The transcription factor Nrf2 (the nuclear factor erythroid 2-related factor 2) presents higher levels by oxidative stress and toxic aggressions. Nrf2 function is the regulation of the expression of numerous proteins that play a role in the antioxidant response, improving the CLL cell survival. The increased mitochondrial mass and the production of mitochondrial ROS activates this signalization pathway in CLL cells [246]. Under normal conditions, Keap1 negatively regulates Nrf2, promoting its degradation through the ubiquitin-proteasome pathway. However, under pathological conditions, such as oxidative stress, Keap1 modified in its cysteine reactive residues, these modifications produce conformational changes in the Keap1 protein, releasing Nrf2. Once released, Nrf2 is translocated to the nucleus and activates the antioxidant and cytoprotective gene transcription [247]. Nrf2 promotes the expression of the catalytic and modulates antioxidant subunits, GCL (Glutamate-Cysteine Ligase) subunits, which enhances the expression of GSH. Furthermore, GSH positively regulates the heme oxygenase-1, which also positively regulates the mitochondrial transcription factor A, stimulating mitochondrial biogenesis. This process reduces the ROS damage and compensates for reduced mitochondrial energy production [248].

Sánchez-Lopez et al. (2020) showed that the activation of p-62-Nrf2 pathway, dependent on NF-kB plays a key role in the survival and drug resistance in CLL cells with high levels of ROR 1, a tyrosine kinase receptor associated with a poor prognosis [249]. The activation of NF-kB by microenvironmental factors such as BAFF (B-cells activation factor), increases ROS production. Furthermore, the signaling adaptor p62 (SQSTM1) is involved in the union of NF-kB with Nrf2. Consequently, higher levels of p62 promote the sequestration of Keap1, protecting the CLL cells by reducing ROS cell effects. In addition, in higher expression of ROR1 CLL cells, the activation of NF-kB through the BAFF signalization improves the autophagy flux, producing an accumulation of p62. To summarize, this process is involved in cell survival and drug resistance, by the reduction in the oxidative stress induced by ROS levels [249].

The autophagy process implications in the disease vary depending on the patient’s stage. In early Binet stage patients, the BECN1 and ATG5 expressions are higher, and LC3-II has shown a similar tendency. These are associated with the del(13q) and the negativity of CD38 biomarker, associating the autophagy process to a better prognosis. Additionally, survival analysis showed that high expression of ATG5 correlated with a longer survival without treatment [250,251]. SLAMF1 is also associated with the prognosis of the disease. Low levels of this gene correlate with aggressive forms of CLL and reduce autophagy. The expression of SLAMF1 depends on the ROS levels within the cell, and a low expression of SLAMF1 negatively regulates ROS, reducing their levels. It also depends on the MAP Kinases that regulate cellular signaling, and by the BCL2 complex phosphorylation, which releases Beclin-1. In conclusion, reduced SLAMF1 levels diminish the formation of autophagy complexes and produce resistance to certain therapies such as fludarabine and ABT-737 [252].

On the other hand, the overexpression of the PI3K components, including the *PIK3C3*, *PIK3R4*, and *BECN* genes, is associated with a poorer prognosis. Additionally, it was verified that these three genes can be independent prognosis markers [253]. Smith et al. (2019) investigated the viability of CLL with autophagy inhibition using VPS34-IN1. They observed that inhibition produces lower levels of LC3B-II mediated for BCR but did not produce effects on BCR signalization. Their study concluded that autophagy has a protector effect in CLL patients, and its inhibition could be a potential therapy [254]. Recently, Chen et al. (2024) showed the role of USF2 in CLL. The overexpression of this gene promotes cell proliferation and inhibits apoptosis, which is related to a poorer prognosis in CLL. Their study revealed that USF2 can act as an autophagy enhancer, since its overexpression produces an increase in the LC3II/LC3I ratio and Beclin-1 expression [255].

Therapeutic implications: In CLL, targeting mitochondrial metabolism offers a promising therapeutic approach, as drugs that disrupt mitochondrial function or increase mitochondrial ROS selectively induce cytotoxicity in CLL cells, sparing normal cells. For instance, PK11195, which targets mitochondrial F1F0-ATPase, increases superoxide production and triggers apoptosis in CLL cells. Additionally, inhibiting antioxidant defenses by targeting pathways such as Nrf2 or STAT3 could reduce the antioxidant capacity of CLL cells, rendering them more susceptible to oxidative damage. Furthermore, combination therapies that pair ROS-inducing agents with inhibitors of autophagy or antioxidant pathways (like p62-Nrf2 and USF2) may enhance therapeutic efficacy and help overcome drug resistance, offering a more effective treatment strategy for CLL.

##### Acute Lymphoid Leukemia

Acute lymphocytic leukemia (ALL) is characterized by the abnormal clonal proliferation of naive or mature T to B lymphocyte cells, leading to their infiltration into bone marrow, peripheral blood, and sometimes other organs and tissues. This disease exhibits significant clinical heterogeneity and diverse biological features. ALL predominantly affects children more than adults, with B-lymphocyte lineage being the most involved subtype [256].

The most common genetic alteration in patients with ALL, occurring in 20–40% of cases, is BCR/ABL translocation [257]. This gene fusion plays a crucial role in cellular growth and the reduction in apoptosis by the transcription of BCR/ABL protein with tyrosine kinase activity [258]. Studies have shown that the BCR/ABL protein can increase intracellular ROS levels through the activation of the NOX complex [259]. Additionally, BCR/ABL can further elevate ROS by activating other pathways, such as the PI3K/AKT/mTOR signaling pathway. Malignant cells with this mutation often develop mechanisms to resist the DNA damage caused by elevated ROS levels [260]. Additional studies analyzed the interaction between the PI3K/AKT pathway and IL-7 in the production of ROS. These studies also demonstrated that the use of ROS eliminators inhibited the viability of T-ALL cells, and in some cases, it induced the death of malignant cells [261]. On the other hand, Lim et al. (2020) discovered that IL-7 signaling in the JAK/STAT pathway enhances cell growth and increases ROS levels in malignant cells. B-ALL cells are dependent on high levels of ROS for survival [262]. NOTCH1, a membrane receptor with an essential function in the proliferation, differentiation, and activation of T-cells, is the least regulated pathway in T-ALL [263]. Patients that carry this mutation have higher levels of ROS due to the regulation of c-Myc that activates the PI3K/Akt/mTOR pathway [264] and the upregulation of CK2 (casein kinase 2) caused by the downregulation of the function of the PTEN protein. The inhibition of CK2 and normal levels of ROS cause the death of T-ALL cells without producing any damage to normal T-cells [265]. Ping et al. (2022) show that the levels of creatine, albumin, or C-reactive protein, indicators of cellular stress levels, could be independent prognostic factors for overall survival (OS) in T-ALL [266].

Kantner et al. (2013) found in murine models that the fusion gene ETV6/RUNX1 (TEL/AML1), the most common chromosomal aberration in the pediatric form of ALL, which occurs in 25% of children with B-ALL, generates a preleukemic clone and induces elevated levels of ROS. These increased ROS levels result in genetic instability and DNA strand breaks, leading to the transformation of preleukemic clones into malignant cells [267]. Polak et al. (2019) discovered another critical function of the aberration ETV6/RUNX1, showing that it regulates autophagy levels in leukemic cells even in the absence of cellular stress. Specifically, ETV6/RUNX1 induces the activation of Vps34, a key component of the central regulatory complex for autophagy. In this context, autophagy promotes the survival and proliferation of leukemic cells. Importantly, the inhibition of Vps34 and autophagy pharmacologically was shown to reduce the survival and proliferation of these cells [268]. Building on this, Bwanika et al. (2024) corroborated the findings of Polak et al. by reporting elevated levels of Vps34 and autophagy in patients with the ETV6/RUNX1 fusion gene. Additionally, they identified the upregulation of ATG14, a protein closely linked to autophagy. These findings emphasize the role of ETV6/RUNX1 in enhancing autophagy and supporting cell survival [256]. Collectively, these studies demonstrate a connection between the ETV6/RUNX1 fusion gene, autophagy, and cellular stress. However, it is necessary to conduct more research in these fields to explore the interplay between these processes and their therapeutics implications.

In B-ALL, resistance to glucocorticoids is the principal treatment for the disease. It is associated with an increased activation of the MAPK pathway, which leads to a poor prognosis. The MEK1/2 inhibitor, selumetinib, enhances the effectiveness of GC and reduces the activation of pERK1/2, also affecting the mTOR pathway [269]. Additionally, selumetinib increases the level of LC3-II, a marker crucial for autophagy [270,271]. In pediatric patients, leukemic cells show low expression of ATGs such as *ATG7*. Additional studies indicated that the deletion of this key gene in murine models resulted in an increased proliferation of leukemic cells [271]. Furthermore, activating autophagy with rapamycin has been shown to improve survival in mice with B-ALL [271]. These findings suggest that targeting autophagy could be a promising therapeutic approach.

In T-ALL, research in Jurkat cells models of the disease, have shown that certain therapies, such as timosaponin A III, can activate autophagy and apoptosis, suggesting that autophagy could be a potential therapy for T-ALL [272]. Another study discovered that the JAK/STAT pathway is frequently mutated in T-ALL, proposing TG101209 inhibitor of JAK2 can suppress the autophagy and the cell proliferation through the modulation of JAK/STAT pathway [273]. Other drugs, like MK-2206, and CQ inhibit the autophagy and protect the malignant cells for the apoptosis [264,274]. In the case of FAPP2, its overexpression in T-ALL is involved in the activation of PI3K/AKT/mTOR pathway contributing to leukemic cell proliferation and survival. The negative regulation of FAPP2 induce the autophagy and trigger the inhibition of the malignant cell proliferation, suggesting that the modulation of the expression of this gene could be a potential therapeutic strategy, due to the autophagy induce by its negative regulation produce a leukemic cell death and help to control the T-ALL progression [264]. Therefore, new therapies with autophagy present challenges and require further investigation, but in general, autophagy suppression represents a potentially interesting therapeutic approach.

Therapeutic implications: Targeting oxidative stress in ALL offers promising strategies for treatment. In T-ALL, ROS eliminators, such as NOX inhibitors or CK2 targeting, can induce apoptosis in malignant cells while sparing normal cells. Combining ROS modulation with inhibitors of critical pathways like PI3K/AKT/mTOR may enhance therapeutic efficacy. In parallel, modulating autophagy provides additional therapeutic potential. Inhibiting autophagy, for example with VPS34 inhibitors, disrupts survival mechanisms in leukemic cells, while activating autophagy with agents like rapamycin can improve outcomes, particularly in B-ALL with low ATG expression. Furthermore, pathway-specific inhibitors targeting PI3K/AKT/mTOR or JAK/STAT pathways can suppress both ROS production and autophagy, reducing leukemic cell viability. Additionally, MEK inhibitors like selumetinib have shown potential in enhancing GC sensitivity while modulating autophagic flux, offering new avenues for therapeutic intervention.

##### Chronic Myeloid Leukemia

Chronic myeloid leukemia (CML) is a malignant myeloproliferative neoplasm characterized by the uncontrolled cell proliferation of myeloid cells in different stages of maturation. The disease progression is heterogeneous, and the patients can present one of three clinical phases: the chronic phase, the accelerated phase and the blast crisis. The chronic phase is the initial stage, defined by less than 10% of blast in bone marrow or peripheral blood. The accelerated phase is an intermediate stage, in which the blast represents between 10 and 19%. Finally, the blast crisis is the most advanced progression, and it is characterized by more than 20% of blast, which could be of myeloid, lymphoid or undifferentiated origin [275].

CML patients have a reciprocal translocation between the long arm of chromosome 9 and the long arm of chromosome 22, resulting in the Philadelphia chromosome (t(9;22)(q34;q11)), which creates the hybrid gene BCR/ABL. This gene encodes a tyrosine kinase with a key function in the transformation of the leukemic HSC, promoting abnormal cellular proliferation, protein synthesis and antiapoptotic signals [276,277]. Nowicki et al. (2004) demonstrated the importance of the aberration BCR/ABL in CML. Their study has shown that the double-strand breaks in the patients with this aberration occur by the increase in ROS levels induced by the gene fusion. Furthermore, the HSC stimulation for growth factors or the BCR/ABL kinase results in higher levels of ROS in comparison than the normal cells [278]. The reason for this is that the Philadelphia chromosome inhibits two detoxifying enzymes, the CAT and Glrx1, contributing to the oxidative stress [279]. Similar to ALL, this aberration can activate the PI3K/mTOR pathway, increasing the intracellular ROS levels [260]. The activation of this pathway induces the activation of ATF5, a transcription factor that regulates mTORC1, depending on Fox4, a factor involved in cell survival and metabolism. This suggests that the BCR/ABL gene increases the expression of mTORC1, contributing to the inhibition of autophagy [280]. On the other hand, studies demonstrated that the inhibitor of BCR/ABL used in the treatment against CML, imatinib, inhibits the expression of microRNA-30a in CML cells producing an increase in autophagic flux and higher levels of the proteins Beclin-1 and ATG5 [281]. In addition, Colecchia et al. (2015) found that MAPK15 (also known as ERK8) plays a crucial role in autophagy induced by BCR/ABL in CML. MAPK15 regulates the interaction between the protein fusion BCR/ABL and the autophagy vesicles, facilitating autophagy activation. It also interacts with the LC3 family proteins depending on LIR (LC3-Interacting region), which is essential for autophagy. The inhibition of MAPK15 reduces cell proliferation and the tumor development produced by the Philadelphia chromosome, presenting MAPK15 as a therapeutic target in CML [282].

Another study in murine models suggests that BCR/ABL kinase activity regulates autophagy by phosphorylating Beclin-1 at tyrosine residues 233 and 352 in CML. This phosphorylation disrupts the interaction between key autophagy regulators, including UVRAG, VPS15, ATG14, VPS34, RUBICON, and Beclin-1. The result is the inhibition of autophagy, which impacts cancer cell survival and proliferation. This mechanism highlights the role of BCR/ABL in manipulating cellular processes to promote leukemia cell survival [269].

Therapeutic implications: In CML, high ROS levels are crucial for cell survival but also contribute to DNA damage. Therapeutic strategies that modulate ROS levels or enhance antioxidant defenses could disrupt this balance, potentially sensitizing CML cells to treatment. Additionally, modulating autophagy presents a promising approach. Inhibiting autophagy in CML cells, for example, by targeting Vps34 or MAPK15, impairs autophagic flux and reduces leukemic cell viability. On the other hand, inducing autophagy in combination with tyrosine kinase inhibitors (TKIs) like imatinib may improve therapeutic outcomes by promoting apoptosis in resistant CML cells. Furthermore, combining TKIs with agents that target oxidative stress or autophagy-related pathways holds promise for improving outcomes in resistant or advanced-phase CML.

##### Acute Myeloid Leukemia

Acute myeloid leukemia (AML) is a hematological malignancy defined by an abnormal growth of myeloid blast or progranulocytes that do not mature properly. The disease has an unfavorable or poor prognosis. In 2024, the estimated new cases are 20,800 (1% of all new cancer diagnoses) and the median age of diagnosis is 69 years. The prognosis is poor with a general survival rate after 5 years lower than 50% in young patients with LMA and lower than 20% in older patients [264].

In AML the ROS levels are essential to predict the prognosis of the patients. There are multiple mechanisms to increase the ROS levels. The mutation in FLT3, affecting 30% of AML patients, is associated with a poor prognosis due to a shorter OS [283]. Stanicka et al. (2015) demonstrated that AML patients carrying this mutation had increased the levels of ROS due to the NOX, specifically NOX4 and p22phox. These molecules act as pro-survival signals [284]. Earlier, Hole et al. (2013) concluded that AML blast with NOX produces higher levels of ROS than the normal blast. They discovered that the ROS produced by NOX2 are associated with dysfunction in p38 MAPK and that inhibiting this molecule improved cell proliferation. Additionally, extracellular ROS contributed to the proliferation of AML cells [285]. More recently, this research group demonstrated that NOX2 enhanced glucose uptake and the glycolysis process through reprogramming cell metabolism. It is produced by the activation of a key enzyme of the glycolysis process, PFKFB3, generating NADPH and biosynthetic precursors in AML [286].

FLT3-ITD (FLT3 tyrosine kinase receptor) triggers downstream pathways such as STAT5, PI3K/AKT, and RAS/MAPK, which are linked to the higher levels of ROS in AML patients [287]. In contrast with other types of leukemias, these higher levels of ROS are cytoplasmic because the FLT3 mutation occurs in the cytoplasmic membrane [283]. Proteins such as Jab1 and TRX, which are involved in cell growth, can be activated by the higher levels of ROS produced by the FLT3 mutation, suggesting that ROS/Jab1/TRX could be a therapeutic target in AML [288]. Rasool et al. (2007) investigated the *NRAS* and *BCL2* genes and the ROS levels in the leukemic cell. Their study in murine models concluded that mutations in NRAS produce higher levels of ROS, increasing cellular stress. Furthermore, the double mutants NRAS and BCL2 produced more ROS levels and had a significant impact on the AML blast [289]. Other authors showed in murine models that autophagy is essential for leukemic initiator cells in the bone marrow but not for the differentiated leukemic blast, as it prevents cellular stress. The accumulation of ROS and mitochondria are closely linked to the maintenance of leukemic initiator cells. When comparing normal and leukemic initiator cells, the latter is shown to have a higher number of mitochondria than the former. In contrast, in peripheral blood, autophagy improves the survival of leukemic cells regardless of their differentiation stage [290]. Additionally, autophagy is closely correlated with glycolysis. Increasing glycolysis levels can suppress autophagy flux producing poorer disease prognosis. Studies show that the inhibition or deletion of the gene ATG5 reduces the levels of autophagy and increases AML cell proliferation by inducing higher levels of glycolysis [291]. Other studies show that the inhibition of ATG3 produces the same effect in tumor progression, showing the importance of autophagy in the disease [292]. Wang et al. (2019) discovered that patients with mutated NPM1 have an increased expression of PKM2, a glycolytic enzyme that increases the phosphorylation levels of Beclin-1, a key molecule in autophagy. They observed that the higher levels of PKM2 are associated with poorer prognosis in AML patients [293]. On the other hand, in de novo AML patients, the basal autophagy flux is lower, and the expression of *ATG7* and *LC3* genes is reduced, showing a strong correlation with autophagy levels. Therefore, a reduction in the autophagy pathway could produce the initiation of leukemogenesis [294].

Patients with *FLT3* mutation are associated with higher levels of basal autophagy, contributing to drug resistance. Elevated autophagy levels are associated with higher expressions of phospho-FLT3, phospho-BKT, and ATF4 in resistant AML cells [295]. Heydt et al. (2017) show in mice that the transcription factor ATF4 depends on FLT3-ITD activity, and the inhibition of ATF4 inhibits the proliferation of AML, increasing survival, mimicking the effects of autophagy inhibition [296]. Recently, Shang et al. (2019) investigated the implication of circular RNA in autophagy in therapy resistance cells. Their study revealed that circPAN3 has an important function in the acquired resistance in AML. circPAN3, which is expressed in resistant AML cells, enhances autophagy levels by the regulation of the AMPK/mTOR pathway, making circPAN3 a new therapy target in relapsed AML [297]. In conclusion, autophagy plays a heterogeneous role in AML. While higher levels of autophagy may improve prognosis due to the inhibition of glycolysis, it may also lead to a worse prognosis due to the resistance of AML cell therapy.

Therapeutic implications: Targeting ROS-producing pathways, such as NOX enzymes or FLT3 signaling, offers a promising strategy to reduce oxidative stress and impair leukemic cell survival. Additionally, the ROS/Jab1/TRX axis has emerged as a potential therapeutic target for disrupting pro-survival signaling in AML cells. Targeting this pathway could enhance the vulnerability of tumor cells to treatment by inhibiting their oxidative defense mechanisms and promoting cell death, offering new avenues for therapy in resistant AML cases. In addition, inhibiting key autophagy regulators, such as ATG5 and ATG7, has shown potential in reducing leukemic cell proliferation by increasing metabolic stress, thereby impairing their survival mechanisms. Emerging therapeutic targets also include circPAN3, which regulates autophagy through the AMPK/mTOR pathway. This circular RNA is implicated in therapy resistance, particularly in relapsed AML, suggesting that targeting circPAN3 could enhance treatment efficacy and overcome resistance in these patients.

#### 6.2.2. Lymphomas

Lymphoma encompasses a diverse group of over 90 subtypes of hematological malignancies, traditionally categorized into Hodgkin lymphoma (HL) and non-Hodgkin lymphoma (NHL). In 2019, these diseases accounted for 4.7% of all newly diagnosed cancer cases in the United States. Known risk factors include genetic predisposition, infectious agents, and inflammatory conditions [298].

##### Hodgkin Lymphoma

HL is the most frequent lymphoma, and the prognosis is generally favorable when using chemotherapy and radiotherapy, as approximately 90% of patients can be cured. HL is usually diagnosed in young adults, around 35 years old. However, whereas chemotherapy is ineffective in some patients, in others, it produces toxic effects and a decrease in life expectancy [299]. Lymphoma is characterized by the presence of abnormal B-cells, Reed–Sternberg (RS) cells, which are big and multinucleated malignant cells, and a high density of immune effector cells in the tumoral microenvironment [300]. The origin of this type of cell is unknown, although Epstein–Barr virus (EBV) could be implicated in their development [301].

Oxidative stress in HL affects RS cells and the surrounding microenvironment. Bur et al. (2014) discovered oxidative stress damage in mononuclear cells of peripheral blood in non-treated HL patients caused by an increase in ROS levels in mitochondria. RS cells suffer from oxidative stress damage in DNA, specifically in advanced stages of HL, which is characterized by an increased expression of 8-OHdG, an oxidative stress marker. This damage produces genomic instability and reduces DNA repair enzymes. However, in aggressive forms of HL, RS cells and the microenvironment produce increased levels of antioxidant enzymes in mitochondria such as Mn-SOD and PrxV. This suggests an adaptive mechanism against oxidative stress in the cells [302]. Later, Marini et al. (2022) validated the presence of oxidative stress in peripheral blood mononuclear cells of untreated patients. Their study proposes that the decoupling of oxidative phosphorylation and the redox stress causes more damage to lymphocytes than to monocytes. The metabolic response in both types of cells involves an increased activity of hexose-6-phosphate dehydrogenase, producing an increase in glucose flux through the ER [303]. These studies suggest that chemotherapy based on increased ROS levels could be failed for the presence of antioxidants in RS and peripheral blood cells. Other studies on the senescence of HL cells revealed that certain senescence pathways are upregulated by oxidative stress. Specifically, oxidative stress increases the expression of p16 INK4a and p21Cip1 producing the inhibition of the cellular cycle in RS cells. Moreover, other biomarkers associated with senescence, such as H2AX and p53, show elevated expression in the Hodgkin lymphoma-derived L428 cell line under oxidative stress condition [304]. Ikeda et al. (2012) studied the HL cell lines L1236 and L428, which were found to have a tumorigenic potential. These cell lines can expel ROS maintaining low intracellular ROS levels. Their study proposed that the population with higher levels of aldehyde dehydrogenase (ALDH) and lower levels of ROS could be cancer initiator cells [305]. Additionally, ROS play a crucial role in the differentiation of the cell types implicated in HL. Immature HL cells reduce ROS levels through the action of HIF-1α, a protein that regulates the cellular response to hypoxic conditions. The stabilization of H1F-1α inhibits the differentiation of the HL cells treated with H_2_O_2_, a ROS that often stimulates cell differentiation. This inhibition is mediated by the protein HO-1, whose primary function is to eliminate ROS [306].

The autophagy process is also involved in the senescence. Some studies have shown that the high expression of p62 in RS cells could indicate a poorer prognosis in patients with HL. The function of p62 is the repair of the nuclear machinery of DNA but, when autophagy is inhibited, the accumulation of p62 inhibits RNF168 producing a reduction in the recruitment of DNA repair proteins. Moreover, this process produces an increase in the DNA damage produced by ROS and the degradation of certain DNA repair proteins [307]. Additionally, EBV appears to influence the autophagy flux levels in HL. In malignant cells, EBV protein LMP1 enhances the autophagy flux modulating stressful situations such as inanition conditions or chemotherapy treatment agents like doxorubicin (DOX). Murine models have shown that the inhibition of autophagy with CQ effectively eliminates HL cells that express LMP1. Interestingly, excessive autophagy can lead to cell death. In HL cell lines like L428 and KM-H2, LMP1 protects against apoptosis and increases the autophagy flux. Nevertheless, an excessive increase in autophagy could produce cell death. Therefore, autophagy acts as a double-edged sword in EBV-associated HL. It can protect tumor cells under certain conditions, but excessively high levels can result in their destruction, presenting autophagy as a promising therapeutic target [308].

Another study investigating the impact of microgravity on autophagy in HL patients found that exposure of human HL cells to time-averaged simulated microgravity (taSMG) for two days led to increased oxidative stress. This effect was attributed to the elevated expression of NOX family genes, while levels of ATPase and ATP synthase were reduced, resulting in lower intracellular ATP levels. Consequently, autophagy was activated via the AMPK/Akt/mTOR and MAPK pathways. However, this autophagy activation was inhibited when cells were treated with the ROS scavenger NAC. The findings suggest that autophagy activation driven by oxidative stress under taSMG conditions could hold potential as a novel anticancer strategy for HL patients [309]. Likewise, Wahyudianingsih et al. (2024) reviewed the role of autophagy in the chemotherapy of HL, and they reported that autophagy is activated in response to DNA damage caused by chemotherapy, which often induces apoptosis in tumor cells. However, in some cases other pathways such as autophagy or senescence could be activated instead of cell death, protecting tumor cells from dying. This process is regulated through the inhibition of mTORC1, ATR/Chk1 signaling, ULK1 phosphorylation, G endonuclease activation, and KU70 protein interaction. In line with previous findings, these results suggested that autophagy inhibition could constitute an efficient therapeutic strategy in HL patients [310].

Therapeutic implications: Inhibiting autophagy with agents such as CQ has demonstrated efficacy in eliminating EBV-positive HL cells by disrupting their adaptive stress responses. Chemotherapy-induced autophagy, on the other hand, can protect tumor cells from apoptosis, highlighting the need to target autophagy-related pathways such as mTORC1 or ATR/Chk1 to enhance treatment efficacy. Additionally, studies in simulated microgravity show increased oxidative stress in HL cells, driven by the upregulation of NOX genes and reduced ATP levels. This stress activates autophagy through the AMPK/Akt/mTOR pathways, suggesting that microgravity could be explored as a novel anticancer strategy for HL.

##### Non-Hodgkin Lymphoma

NHL is the most common hematological malignancy, and it is characterized by a proliferation of different B and T cells. It is differentiated from HL by the absence of RS cells and the histology markers CD15 and CD30. It is a very heterogeneous disease with more than 40 different subtypes [311]. Oxidative stress, which arises by an imbalance between pro-oxidant and antioxidant mechanisms, plays a crucial role in NHL. Wang et al. (2006) highlighted the importance of this pathway in NHL by the genotyping of 13 single nucleotide polymorphism (SNPs) in 10 genes of the oxidative stress pathway including *AKR1A1*, *AKR1C1*, *CYBA*, *GPX*, *MPO*, *NOS2A*, *NOS3*, *OGG1*, *PPARG* and *SOD2*. They concluded that the *NOS2A*, *SOD2* and *PPARG* genes could play a role in the oxidative stress and the risk of developing NHL [312]. Subsequently, Lan et al. (2007) analyzed 10 candidate genes from oxidative stress pathway (*AKR1A*, *AKR1C1*, *AKR1C3*, *CYBA*, *GPX1*, *MPO*, *NOS2A*, *NOS3*, *OGG1* and *SOD2*) in a cohort of female patients and identified 14 SNPs within the *NOX*, *AKR1A1* and *CYBA* genes significantly associated with the risk of developing NHL [313]. Likewise, Gustafson et al. (2014) studied polymorphisms in 28 genes of the oxidative stress pathway in NHL patients treated with anthracycline-based therapies. Their study identified that homozygous patients for the rs188312 SNP within the *NCF4* gene could be involved in the treatment outcomes because these patients showed a higher risk of hematological toxicity [314].

The autophagy process has been implicated in several types of NHL. For instance, chLym-1, a monoclonal anti-HLA-DR antibody, can activate the autophagy process in Raji cells, a cell line derived from an NHL subtype (Burkitt lymphoma). In treated patients, chLym-1 acts by inducing apoptosis through the activation of autophagy pathways such as Akt/mTOR and MEK/Erk [315]. In the case of mantle cell lymphoma (MCL), the association between TG2 and IL6 activates autophagy, promoting MCL cell survival. Moreover, the interaction with ATG5 produces a positive regulation of TG2/NFκB/IL6 signaling [316]. In primary effusion lymphoma (PEL), the anti-tumoral effects of CQ inhibited the autophagy process. This inhibition produced the accumulation of unfolded proteins, producing ER stress. These conditions induced apoptosis in PEL cells, suggesting that autophagy inhibition could be a potential therapy for PEL patients [317]. Considering these results, it seems that the role of autophagy in NHL is heterogeneous and varies according to the disease subtype.

Therapeutic implications: In NHL, autophagy acts as a double-edged sword in therapy; while it supports tumor cell survival under stress, its excessive activation can lead to cell death. Targeting autophagy pathways offers a promising strategy to enhance treatment efficacy or overcome drug resistance. Modulating oxidative stress pathways, such as reducing ROS levels or targeting antioxidant defenses, could also disrupt tumor survival mechanisms and increase the effectiveness of treatment. Additionally, manipulating autophagy by either inhibiting protective autophagy or inducing excessive autophagy could potentiate therapeutic responses and improve disease outcomes.

##### Diffuse Large B-Cell Lymphoma

Diffuse large B-cell lymphoma (DLBCL) is the most common type of lymphoma, accounting for approximately 30% of all cases. It is an aggressive form of B-cell lymphoma, with an average age of diagnosis around 70 years. The primary treatment typically involves chemotherapy, often combined with immunotherapy, including options such as chimeric antigen receptor T-cell (CAR-T) therapy for refractory or relapsed cases [318]. Nakamura et al. (2022) investigated oxidative stress as a prognosis factor in untreated patients with DLBCL. They showed that oxidative stress levels were significantly higher in patients compared to healthy controls. Derivatives of reactive oxygen metabolites correlated with several prognosis factors, including sIL-2r, a biomarker associated with the lymphoma activity, the international Prognostic Index that evaluates the risk of DLBCL and with elevated levels of lactate dehydrogenase that is linked with metabolic activity and tumor proliferation. The study concluded that oxidative stress may be associated with poorer prognosis, and that it plays an important role in the carcinogenesis of DLBCL patients [319]. Additional studies have consistently underscored the importance of the glutamine metabolism in DLBCL. In DLBCL, glutamine metabolism is upregulated, producing elevated levels of glutamine and lower levels of α-KG. Through the activity of malate dehydrogenase 1, α-KG is converted into 2-hydroxyglutarate, resulting in elevated levels of ROS in tumor cells. High ROS levels induce ferroptosis by activating lipid peroxidation and enhanced TP53 expression, which is associated with DNA damage. Furthermore, dimethyl-α-ketoglutarate inhibits tumor proliferation, suggesting that the regulation of glutamine metabolites could constitute a new therapy for DLBCL [320].

On the other hand, Zhao et al. (2025) studied the role of some oxidative stress-related genes in DLBCL. They identified 26 genes that were crucial for tumor proliferation processes such as DNA damage, lipid peroxidation, and the escape of the immune system. Notable genes included *CCND1*, *GPX3*, *ICAM1*, *IFNG*, *MT2A*, *NDRG1*, *NLRP3*, *PLAU*, *SQSTM1* and *TXN*. These researchers demonstrated that patients could be classified into two groups based on differences in immunity infiltration that were dependent on the levels of oxidative stress. The infiltration of tumor-killing cells, including CD4/CD8 T cells, dendritic cells, macrophages, and NK cells, differed significantly between groups. These differences were accompanied by markedly distinct levels of oxidative stress, which were likely responsible for the observed immune disparities [321]. Like HL, elevated levels of certain biomarkers, such as γH2AX and 8-OHdG, were associated with aggressive subtypes of DLBCL, particularly those positive for MYC/BCL2, including the Activated B-cell (ABC) subtype and high-grade B-cell lymphoma (HG-BCL). In these subtypes, the activation of DNA repair mechanisms and increased BCL-2 expression enable cells to withstand the oxidative stress induced by the oncogenic activity of MYC. Based on this observation, targeting DNA repair mechanisms and BCL2 inhibition could alleviate oxidative stress in malignant cells and enhance apoptosis without relying on conventional chemotherapy [322]. Prior to this study, Mai et al. (2016) investigated the role of oxidative stress in the two main subtypes of DLBCL: activated B-cell-like (ABC-DLBCL) and germinal center B-cell-like (GCB-DLBCL). ABC-DLBCL is more resistant to treatment, and the effectiveness of doxorubicin (DOX) in this subtype depends on its ability to generate reactive oxygen species (ROS) to kill tumor cells. In contrast, GCB-DLBCL is more sensitive to chemotherapy, where DOX primarily induces DNA damage through the activation of DNA repair mechanisms.

In the ABC-DLBCL subtype, activation of the STAT3 protein is a key feature. STAT3 regulates antioxidant mechanisms, including the expression of the SOD2 enzyme, which neutralizes ROS and contributes to the resistance of malignant B cells to DOX. However, when ROS levels exceed a critical threshold, STAT3’s capacity to mitigate oxidative stress collapses, leading to cell death. This makes STAT3 a potential therapeutic target for DLBCL [323]. Additionally, evidence suggests that STAT3 plays a role in autophagy by suppressing oxidative stress-induced autophagy and protecting mitochondria from mitophagy [324]. Further studies have explored STAT3 inhibition in the context of antiretroviral therapy, which inhibits cellular proliferation and induces apoptosis, autophagy, and ferroptosis. These findings indicate that STAT3 inhibition is essential for regulating therapy, and combining antiretroviral therapy with autophagy inducers or STAT3 inhibitors could offer a novel treatment strategy for DLBCL [325].

Concerning autophagy, Li et al. (2019) investigated the role of *CUL4B*, a gene associated with autophagy and involved in multiple types of cancer, in the DLBCL. Their study showed that *CUL4B* is overexpressed in DLBCL and contributes to characteristics of aggressive tumors, such as a larger tumor size, metastasis and poorer prognosis. CUL4B regulates certain signalization pathways such as JNK that regulates several cellular processes including autophagy. Specifically, CUL4B positively regulates the activity of JNK, thereby promoting the autophagy process. Taking this into account, the inhibition of CUL4B could serve as a potential therapeutic target by inhibiting the JNK pathway, reducing the autophagy process, and ultimately reducing cell survival [326]. Other studies have developed a prognostic model based on the *ADD3*, *IGFBP3*, *TPM1*, *LYZ*, *AFDN*, *DNAJC10*, *GLIS3* and *CCDC102A* genes, which are involved in autophagy. This model integrates genomic prediction and immunological infiltration, offering a new therapeutic tool in personalized medicine, as they permit prediction of the survival probability and the drug resistance [327]. In addition, Mandhair et al. (2024) emphasized the pivotal role of ULK1, a key protein in the autophagy process, in germinal center B-cell-like diffuse large B-cell lymphoma (GCB-DLBCL). They found that ULK1 was overexpressed in patients with this disease subtype and influenced their response to treatment. Their findings suggest that suppressing ULK1 could represent a therapeutic strategy for GCB-DLBCL. Additionally, the study proposed that ATG biomarkers might serve as predictors of treatment response [328]. Another gene significantly influencing autophagy and DLBCL is *BECN1*, which encodes Beclin-1 protein. Autophagy activation associated with Beclin-1 contributes to improved prognoses by overcoming acquired resistance and enhancing therapeutic outcomes. Notably, venetoclax, which disrupts the Beclin-1/BCL2 interaction, has shown potential to induce autophagy and improve the efficacy of chemotherapy in treating DLBCL [329].

Therapeutic implications: Targeting oxidative stress presents a promising strategy for improving outcomes in DLBCL. Reducing ROS levels could alleviate DNA damage and inhibit tumor proliferation, while exploiting the heightened sensitivity of malignant B cells to elevated ROS could enhance cytotoxicity. In the ABC-DLBCL subtype, targeting antioxidant mechanisms like STAT3 or promoting excessive ROS accumulation may help overcome resistance to therapies like doxorubicin. For MYC/BCL2-positive subtypes, inhibiting DNA repair mechanisms or targeting BCL2 could enhance apoptosis by disrupting the oxidative stress balance. Therapeutic strategies such as combining STAT3 inhibitors with autophagy modulators or antiretroviral therapies have shown potential to induce apoptosis and ferroptosis in resistant DLBCL cases. Combination therapies that pair chemotherapy with agents modulating redox homeostasis and autophagy may improve therapeutic efficacy and reduce resistance, offering a more effective approach to treating DLBCL.

## 7. Therapeutic Potential

Autophagy and oxidative stress are intricately linked processes with significant implications for cancer therapy. Oxidative stress, driven by ROS, modulates autophagy through key signaling pathways such as AMPK, MAPK, Akt, and JNK, thereby influencing cancer cell survival, proliferation, and stress adaptation [330,331,332,333,334]. At low to moderate levels, ROS act as signaling molecules to activate these pathways, whereas excessive ROS levels induce autophagy as a protective mechanism [335]. Autophagy plays a dual role in cancer: it suppresses tumorigenesis by removing damaged organelles and mitigating oxidative damage [336], but it also enables tumor survival under conditions such as hypoxia, starvation, and therapeutic stress, contributing to drug resistance [337,338].

Therapeutic strategies targeting autophagy are promising but complex. General autophagy inhibition by agents such as CQ and HCQ has shown potential in overcoming resistance, although its efficacy varies with cancer type and treatment context [338,339]. In addition, selective types of autophagy, such as mitophagy and lysophagy, are emerging as precise tools for therapy, offering avenues to disrupt cancer-specific mechanisms [14,340]. ROS-inducing therapies, including chemotherapy and radiotherapy, exploit the dynamic interplay between oxidative stress and autophagy to improve treatment outcomes, although careful modulation is required to prevent resistance [341,342]. The dual role of autophagy and oxidative stress in cancer biology highlights their therapeutic potential as targets for innovative cancer therapies.

### 7.1. Autophagy Modulators

Targeting autophagy is a promising approach for cancer therapy. Below, we report key strategies organized by therapeutic focus:

#### 7.1.1. Role of Autophagy Inhibitors

Autophagy inhibitors have emerged as important tools in cancer therapy, enhancing the efficacy of conventional treatments by sensitizing cancer cells. Agents like 3-methyladenine (3-MA), wortmannin, CQ, and HCQ have demonstrated promising effects in hematological malignancies and solid tumors affecting cancer cell viability, whereas wortmannin has shown to inhibit autophagy independently of nutrient availability and promote apoptosis by downregulating proliferative pathways (PI3K/Akt and NF-kappaB) [338,343,344].

Clinically approved CQ and HCQ, which block lysosomal fusion, not only enhance chemotherapy efficacy in leukemias and lymphomas [21,64] but also exhibit anticancer effects beyond autophagy suppression and promote drug sensitization in both solid tumors and hematological malignancies [21,64,345,346,347,348]. However, the relatively limited potency of these agents has driven the development of more potent analogs, such as EAD1, which has shown encouraging preclinical results in solid tumors [349,350]. These findings underscore the therapeutic potential of autophagy inhibitors while highlighting the need for further optimization to improve potency and specificity. In addition, autophagy modulation through targeted therapies offers new opportunities in cancer treatment. Tyrosine kinase inhibitors like imatinib, INNO-406, and dasatinib induce autophagic cell death in CML and ovarian cancer, demonstrating the utility of leveraging autophagy as a cell death mechanism [349]. mTOR inhibitors such as rapamycin and its analogs (temsirolimus, everolimus, and deforolimus) stimulate autophagy and exhibit anti-tumor activity in multiple hematological malignancies including AML, MCL, and MM [351,352,353], while AMPK activators like metformin [354] and AICAR [355] suppress proliferation and induce apoptosis through autophagy activation among other mechanisms [349,354,356,357]. Additionally, the modulation of pathways such as Akt, mTOR, and tyrosine kinases, as well as other key signaling pathways like Notch, Wnt, and Hedgehog, underscores the complexity and context-dependent roles of autophagy in hematological malignancies [358,359,360] and solid tumors [361,362]. These approaches highlight autophagy’s dual potential to either inhibit tumor initiation or promote cancer progression, depending on the cancer type and therapeutic context, offering diverse strategies for cancer management.

#### 7.1.2. ATGs and Proteins

ATGs and proteins play pivotal roles in cancer progression and therapy, acting as critical modulators of tumorigenesis and cellular survival. Mutations in ATGs, such as *ATG2B*, *ATG5*, *ATG7*, *ATG9B*, and *ATG12*, have been linked to frameshift mutations in leukemias [363] but also gastrointestinal and liver cancers [364], highlighting their significance in cancer biology. Similarly, Beclin-1, a key regulator of autophagosome formation, often shows allelic loss, reduced or increased expression [329,365] or inhibiting phosphorylation in hematological malignancies [365] and solid tumors, implicating its dysfunction in carcinogenesis [366].

Other players, such as p62 (SQSTM1), which activates tumor-promoting NFκB and Nrf2 pathways [367], and mitophagy receptors BNIP3 and BNIP3L (NIX), which protect against tumorigenesis by maintaining mitochondrial quality [368], further demonstrate the multifaceted role of autophagy in hematological malignancies [369,370,371] and its role in disease prognosis [371].

These findings underscore the intricate functions of autophagy-associated pathways in regulating tumor growth and survival. The diverse roles of these genes and proteins not only deepen our understanding of cancer biology but also reveal promising targets for therapeutic development, paving the way for novel interventions in cancer treatment.

#### 7.1.3. Flavonoid-Based Autophagy Modulation

Flavonoids, a diverse group of plant-derived compounds, have received considerable attention for their anticancer potential, largely due to their ability to modulate autophagy. Compounds such as apigenin, quercetin, epigallocatechin gallate (EGCG), and curcumin exhibit potent biological activity in hematological malignancies despite challenges related to their limited oral bioavailability [372,373].

Clinical studies underline their therapeutic relevance. For example, a bioflavonoid mixture containing apigenin and EGCG (20 mg each) is currently being studied as a preventive measure against recurrence in hematological malignancies and solid tumors, highlighting its translational potential in both hematology and oncology areas [374]. In hematological malignancies, flavonoids have been found to interfere with different signaling pathways and molecules, demonstrating anticancer properties in leukemia and lymphoma cells [375,376]. In addition, it has been found that flavonoids induce cell cycle arrest, apoptosis, inhibition of fatty acid synthesis, oxidation, and metal chelation, and they have chemosensitization features [377,378]. These results suggest that the integration of flavonoids with traditional chemotherapy agents might constitute a promising therapeutic approach. In line with this hypothesis, it has been reported that the use of quercetin or flavonoid methyl esters in combination with specific mitogen-activated extracellular kinases (MEK) 1/2 inhibitors substantially enhanced leukemic cell death, confirming the clinical implications for the use of these compounds in combination with MEK 1/2 inhibitors as potential therapeutic agents for leukemia [376]. Additionally, it has been demonstrated that flavonoids such as quercetin, catechin, and brusatol reduce the risk of lymphoma [379] by inhibiting proliferation and inducing the apoptosis of tumor cells. Similar effects have been observed in ALL, AML, CLL, CML, and MM cell lines [372,373,380,381]. Importantly, they are also able to induce apoptosis and promote tumor regression in lymphoma and myeloma xenograft models acting synergistically with dexamethasone, venetoclax, or bortezomib [382,383,384,385]. However, other authors claim that caution should be taken with their use as flavonoids because it could inhibit the anticancer effects of bortezomib [386]. Curcumin, another prominent flavonoid, has demonstrated safety and efficacy in a range of hematological malignancies [387], further validating its clinical applicability [388]. Curcumin diminishes the viability and survival rate of leukemia, myeloma, and lymphoma cells by inducing cell cycle arrest and apoptosis, and it inhibits molecular pathways linked to tumor progression such as NFKB, STAT, Akt/PI3K, and MEK/ERK [387,389,390,391]. The use of Curcumin in a myeloma patient with a third relapse and in the absence of further anti-myeloma treatments, controlled the disease for 5 years with good quality of life [390]. In addition, it has been suggested that Curcumin enhances the efficacy of chemotherapy drugs by modulating drug resistance pathways [387] and might represent a viable alternative to corticosteroids in combination with immunomodulatory drugs or proteasome inhibitors [392]. Similarly, Silibinin shows promise as a therapeutic intervention for β-Talassemia, AML, anaplastic large cell lymphoma, and MM [393]. However, despite the large amount of information available, the mechanistic effects of flavonoids on autophagy are nuanced, as they can stimulate or inhibit autophagic pathways depending on the context. Compounds such as EGCG and quercetin play dual roles in regulating cellular processes such as cell survival, angiogenesis, and resistance to therapy. While some flavonoids, such as silibinin, induce toxic autophagic cell death, which contributes to their anti-tumor effects, others may promote tumor survival by activating protective autophagy, thereby complicating their therapeutic impact [394,395]. These findings suggest that the flavonoid-induced modulation of autophagy holds promise as a multilayered approach to cancer therapy, which requires further investigation to optimize its clinical benefits.

#### 7.1.4. Targeting ROS via Autophagy

Keeping ROS levels low is essential for normal hematopoiesis and stem cell function, and impaired ROS homeostasis is a common signature of hematological malignancies, such as AML and CML [396]. In addition, chronic oxidative stress has been associated with BCR-ABL, FLT3-ITD, and RAS mutations; genomic instability and DNA damage; and disease relapse and poor prognosis in AML patients [397]. On the other hand, given that ROS play a central role in the regulation of autophagy, several chemotherapeutic agents have exploited this interplay to enhance their efficacy in cancer treatment [398]. For instance, arabinocytosine (Ara-C), a purine analog used as a first-line treatment in AML (also known as cytarabine), has been found to induce ROS production, which in turn can trigger autophagy in leukemic cells. Interestingly, enhanced autophagy has been observed in AraC-resistant U937 leukemia cells, suggesting a potential role of ROS-induced autophagy in cancer cell survival [399,400] and drug resistance [400]. In addition, Ara-C reduced the phosphorylation of mTOR and its downstream target p70S6 kinase in REH cells, which was associated with the downregulation of the mTOR activator Akt and the activation of extracellular signal-regulated kinase. These data suggested that the therapeutic efficiency of Ara-C in leukemic patients could be increased by the inhibition of the mTOR-dependent autophagic response [399,401]. Similarly, leukemic cells treated with anthracyclines exhibited increased ROS formation and enhanced autophagy, which promoted tumorigenesis and drug resistance [402]. However, in other cases, autophagy contributed to cytarabine’s antineoplastic effects, particularly at low doses [403], which suggest a complex and dual effect of autophagy in blood cancers. While the precise mechanisms of this dual effect remain to be elucidated, it highlights the promise of targeting autophagic pathways in blood cancer treatments.

#### 7.1.5. Antidepressants as Autophagy Modulators

Antidepressants have emerged as interesting modulators of autophagy in cancer, exhibiting both stimulatory and suppressive effects depending on the type and stage of the disease. Tricyclic and tetracyclic antidepressants (TCA/TeCAs) such as imipramine, desipramine, and amitriptyline have been investigated for their role in autophagy regulation. Maprotiline has shown the ability to induce autophagic programmed cell death in chemoresistant Burkitt lymphoma cells, highlighting its potential against resistant cancers [348]. Similarly, selective serotonin reuptake inhibitors (SSRIs) have shown anti-tumor activity through their effects on autophagy. For example, sertraline acts through both apoptotic and autophagic pathways and has potent effects in acute myeloid leukemia cells [404,405]. In addition, loss of the selective autophagy receptor p62 impaired murine myeloid leukemia progression and mitophagy, which suggested that antidepressants have potential in modulating autophagy and exhibiting anticancer effects in hematological malignancies [406]. On the other hand, Vortioxetine has been shown to induce apoptosis and autophagy in gastric cancer cells via the PI3K/AKT pathway, representing a novel therapeutic approach for this solid tumor. Likewise, paroxetine was found to block autophagic flux and cause mitochondrial fragmentation in lung cancer cells, illustrating a unique mechanism of action [348]. These examples highlight the potential of antidepressants, including TCAs, TeCAs, and SSRIs, as modulators of autophagy, offering innovative strategies for therapeutic intervention in hematological malignancies and solid tumors.

### 7.2. Selective Autophagy Processes as Therapeutic Targets

Mitophagy, the selective degradation of damaged mitochondria, is a therapeutic target in cancer treatment with several promising compounds. For example, BH3 mimetics targeting different BCL-2 family members have been found to be efficient at killing AML cells through the activation of the apoptosis pathway [407]. Interestingly, blockage of autophagy or specific targeting of MFN2 potentiates BH3-mimetic action in eliminating leukemic cells [407]. Likewise, there has been reported that splicing factor mutations (SRSF2P95H/+) are common in hematological malignancies (MDS and AML) and that the inhibition of splicing with glycogen synthase kinase 3 inhibitors impairs mitophagy and activates apoptosis in SRSF2P95H/+ mutated cells [408]. These results suggest that combining mitophagy inhibitors with anticancer agents could represent an effective approach to overcome drug resistance in cancer [409]. Some natural compounds have been shown to affect cancer cell death and exhibit anticancer properties by modulating mitophagy [410]. Notably, fluorizoline inhibits mitophagy by targeting PHB1/PHB2, disrupting mitochondrial energy production and demonstrating anti-tumor effects in hematological malignancies [411,412]. Additionally, fluorizoline upregulates pro-apoptotic factors such as NOXA and BIM, inhibits C-RAF activation, and increases p21 expression, thereby exhibiting activity against CLL, CML, and AML cells [411,412]. Importantly, fluorizoline shows anti-tumoral activity in CLL irrespective of *TP53* and *ATM* gene alterations or *IGHV* mutation status [411]. However, unlike ibrutinib, it failed to prevent leukemia development in a mouse model of aggressive CLL [413]. Moreover, while no studies to date have investigated its effects in hematological malignancies, nitazoxanide has been reported to promote ROS-mediated mitophagy in solid cancers and exhibits synergistic effects when combined with CQ, a well-established autophagy inhibitor [414].

Besides mitophagy, ER-phagy plays a critical role in cancer therapy as it is regulated by the ubiquitin–proteasome system and autophagy. Loperamide induces ER-phagy and potently inhibits the proliferation of leukemia cell lines and primary leukemia cells from AML and ALL patients in a dose-dependent manner [415]. In addition, it triggers DNA damage and induces apoptosis in leukemic cells [415]. Additionally, xenophagy, the autophagic degradation of intracellular pathogens, is another key therapeutic mechanism in hematological malignancies. Resveratrol has been demonstrated to have anti-proliferative and pro-apoptotic effects in various leukemic cell lines by inducing autophagy through AMPK activation and JNK-mediated p62/SQSTM1 expression [416], and inhibiting PI3K phosphorylation and Akt/mTOR pathway, reducing cyclin D1, and upregulating Caspase-3 [417,418]. However, its use in clinical trials has shown unexpected results. A clinical trial using SRT501, a formulation of resveratrol, in MM patients was terminated due to adverse events, including renal failure [419]. Additionally, salinomycin exhibits potent inhibitory activity against AML and mixed lineage leukemia-rearranged (MLLr) cell lines and primary cells [420] and impairs colony formation and reduces leukemia repopulation ability in AML and MLLr models [420]. Finally, lipophagy, the selective degradation of lipid droplets, has also emerged as a valuable target in cancer therapy. Tripterine (celastrol), a novel HSP90 inhibitor, activates lypophagy and it has been shown to inhibit proliferation of leukemia cells, including acute promyelocytic leukemia (APL) HL-60 cells. It depletes Bcr-Abl and induces apoptosis in imatinib-resistant CML cells harboring T315I mutation [421]. Furthermore, celastrol induces cell apoptosis and inhibits the expression of the AML1-ETO/C-KIT oncoprotein in t(8;21) leukemia [422]. Notably, celastrol has also been suggested as an effective therapeutic agent in signal transduction therapy for the treatment of patients with MM. It induces cell cycle arrest at G1 phase and apoptosis in human myeloma U266 cells through the activation of caspase-3 and NF-κB pathways [423,424,425]. Finally, it has been demonstrated that celastrol has synergistic effects with other drugs. For instance, it enhances the cytotoxic effects of TNF, paclitaxel, and doxorubicin in leukemia cells [426].

Finally, lysophagy, the degradation of damaged lysosomes, is targeted by compounds such as loperamide and pimozide, which induce lysosomal membrane permeability, leading to apoptosis of cancer cells [415]. Pimozide also inhibits STAT5, exhibiting efficacy in models of AML driven by FLT3 mutations [427]. These findings illustrate the therapeutic promise of targeting specific forms of autophagy to treat different types of hematological malignancies.

### 7.3. Antioxidant Therapies

Antioxidant therapies based on oxidative stress in cancer exploit the susceptibility of cancer cells to elevated levels of ROS. Here are the main types and their mechanisms of action:

#### 7.3.1. Pro-Oxidant Chemotherapeutic Agents

Pro-oxidant chemotherapeutic agents play a crucial role in the treatment of hematological malignancies by inducing oxidative stress to enhance their anticancer efficacy [428] and even help in designing individualized therapies for patients suffering from refractory diseases [429]. Cisplatin, for instance, exerts its effects by binding to the N7 position of guanine in DNA, interfering with repair mechanisms and preferentially targeting guanine over adenine [430]. This binding promotes the overproduction of ROS, reducing the antioxidant defenses of cancer cells, which in turn increases DNA damage and enhances cisplatin’s overall anticancer activity [431,432]. These combined effects make cisplatin a potent pro-oxidant therapy for several cancers, including hematological malignancies. Cisplatin inhibits cell proliferation and induces apoptosis in APL cells by forming DNA adducts and by activating p53 and AP-1 transcription factors [433]. Similarly, anthracyclines such as doxorubicin targets DNA replication and repair by intercalating into replicating DNA and inhibiting topoisomerase II [434]. In addition to disrupting these processes, anthracyclines generate oxygen-derived free radicals through two mechanisms: a non-enzymatic pathway involving iron and an enzymatic pathway associated with the mitochondrial respiratory chain. Both pathways contribute to oxidative damage, thereby enhancing the therapeutic efficacy of anthracyclines [431]. These dual mechanisms highlight the potential of pro-oxidant chemotherapeutic agents in exploring oxidative stress to combat hematological malignancies [435]. However, despite the promise of pro-oxidative therapies, challenges remain in achieving the selective targeting of malignant cells while sparing normal hematopoietic cells. One potential strategy to address this issue could be combining pro-oxidant agents with other treatments to improve therapeutic outcomes.

#### 7.3.2. Small Pro-Oxidant Molecules

Elesclomol (STA-4783), imexon, motexafin gadolinium (MGd), and buthionine sulfoximine (BSO) are pro-oxidant agents that exploit oxidative stress to promote cancer cell death. Elesclomol chelates copper ions and transports them into mitochondria, disrupting the mitochondrial respiratory chain and inducing apoptosis. Imexon and MGd enhance oxidative stress by inhibiting the antioxidant defenses of cancer cells, while BSO targets the glutamate-cysteine ligase complex, a key enzyme in GSH synthesis. By reducing GSH levels, BSO further increases cancer cell susceptibility to oxidative damage, highlighting the therapeutic potential of pro-oxidant strategies in cancer treatment [431]. In AML, elesclomol has shown a potent anti-leukemic effect at concentrations as low as 10 nM, which is well below the concentrations achieved in cancer patients [436]. In addition, imexon induced apoptosis in MM tumor cells [437] and has shown to have efficacy in clinical trials for MM [438] and refractory B-cell non-Hodgkin lymphoma [429]. Likewise, MdG induces oxidative stress by oxidizing intracellular metabolites, leading to the generation of ROS and apoptosis in malignant cells, including those from CLL, non-HL, and MM [439,440]. Interestingly, preclinical studies have reported that MGd is cytotoxic to various hematological malignancies. It has been shown to enhance the effects of rituximab in NHL and has induced complete remissions when combined with radioimmunotherapy in relapsed NHL patients [439]. Similarly, BSO synergistically enhances melphalan activity against MM [441], whereas elesclomol in combination with paclitaxel showed improved efficacy compared to paclitaxel alone, particularly in terms of progression-free survival in patients with metastatic solid tumors [442,443]. These results point out that parallel strategies need to be explored for hematological malignancies for all these pro-oxidant compounds.

#### 7.3.3. Targeted Therapies

NOX inhibitors and GSH depletion are strategies that modulate oxidative stress to target cancer cells. NOX inhibitors reduce ROS production by targeting NOX enzymes overexpressed in certain cancers. For instance, NOX2 is critical for the self-renewal and differentiation of leukemia-initiating stem cells (LSCs) and its inhibition impairs core metabolism in LSCs, leading to reduced disease development in murine models of leukemia [444]. This suggests that NOX2 plays a significant role in maintaining the malignant phenotype of LSCs, making it a potential therapeutic target for hematological cancers. Likewise, several studies have reported that GSH depletion improves the therapeutic effects of drugs by increasing oxidative stress within cancer cells, making them more susceptible to treatment [445]. Together, these approaches highlight the therapeutic potential of manipulating oxidative stress pathways in the treatment of hematological malignancies.

### 7.4. Approaches Combining Oxidative Stress and Autophagy

Combination therapies targeting autophagy and oxidative stress in cancer have shown significant promise in preclinical studies, leveraging their intricate interplay to enhance therapeutic efficacy. Autophagy, by clearing dysfunctional mitochondria, reduces ROS accumulation and protects leukemia cells from oxidative stress [446]. Research by Sumitomo et al. revealed that leukemia-initiating cells lacking autophagy, due to the deletion of ATG5 or ATG7 in AML mouse models, exhibited increased mitochondrial activity and higher ROS levels [290]. This led to enhanced cell death, underscoring the essential role of autophagy in supporting leukemia-initiating cell survival [290]. Therefore, combining pro-oxidants with chemotherapy, such as nutrient deprivation paired with anticancer therapies, further increases ROS production and promotes apoptosis in cancer cells [134]. A recent study showed that caloric and nutrient restriction during chemotherapy for B-cell ALL reduced minimal residual disease (MRD) risk, suggesting improved treatment efficacy [447]. In addition, other studies have shown that combining pro-oxidants with chemotherapy, such as nutrient deprivation paired with cisplatin or methioninase (a methionine-depleting enzyme), further increases ROS production and promotes apoptosis in cancer cells [448,449,450]. Moreover, AML blasts—malignant cells with significant deficiencies in the arginine-recycling pathway—have been found to be sensitive to BCT-100, a pegylated human recombinant arginase. BCT-100 induces a rapid depletion of both extracellular and intracellular arginine levels, leading to the inhibition of AML blast proliferation and a reduction in AML engraftment [451]. Interestingly, BCT-100 acted synergistically in combination with cytarabine [451]. Additionally, targeting specific proteins and pathways, such as H_2_O_2_-activated AMPK or p62 oxidation, offers novel avenues for therapy [140]. Strategies that inhibit antioxidant enzymes like GPXs can help in predicting disease outcome and overcome drug resistance by increasing oxidative stress and sensitizing tumors to treatment [452]. These approaches demonstrate the potential of combining autophagy modulation with oxidative stress therapies, either by suppressing autophagy’s pro-survival role or enhancing its tumor-suppressive effects, tailored to cancer type and genetic context [134,349].

## 8. Future Directions, Current Limitations, and Emerging Technologies and Approaches

### 8.1. Future Directions

Personalized approaches are crucial for advancing cancer therapies by tailoring autophagy and oxidative stress modulation to the unique characteristics and genetic profiles of individual tumors. Such customization could enhance therapeutic precision and improve patient outcomes. Combination therapies represent another promising avenue, focusing on the synergistic effects of pairing autophagy modulators with traditional chemotherapies or targeted therapies. These strategies may boost treatment efficacy by leveraging complementary mechanisms of action. Biomarker identification is vital for the prediction and monitoring of therapy responses. Discovering reliable biomarkers for autophagy and oxidative stress-based treatments could help refine patient selection and track therapeutic effectiveness more accurately. Novel drug discovery is also a key area of focus, aiming to identify new compounds capable of selectively modulating autophagy or oxidative stress pathways in cancer cells. These targeted interventions could minimize off-target effects and improve treatment specificity. Improved mechanistic knowledge is essential to deepen our understanding of the molecular interplay between autophagy, oxidative stress, and cancer progression. Such insights can uncover new therapeutic targets and inform the design of innovative treatments. Optimizing treatment timing is another critical consideration, as the therapeutic benefit of autophagy modulation may depend on its timing relative to cancer type and stage. Determining the ideal timing could enhance treatment efficacy and reduce resistance. Finally, exploring the tumor microenvironment is necessary to understand how autophagy and oxidative stress influence cancer progression and treatment response within this complex ecosystem. Investigating these dynamics could reveal novel strategies to disrupt tumor growth and improve therapeutic outcomes.

To realize the potential of autophagy modulation in cancer therapy while reducing risks and improving patient outcomes, future research directions should focus on overcoming these challenges.

### 8.2. Current Limitations in Research and Clinical Implications

#### 8.2.1. Research Limitations

The regulation of autophagy constitutes a key obstacle to the development of targeted cancer therapies. The intricate link between autophagy and oxidative stress in cancer cells remains poorly understood, complicating the development of effective therapeutic strategies [129,453]. Additionally, autophagy can act as both a tumor suppressor and a tumor promoter depending on cancer type, stage, and genetic factors, further complicating the development of universal therapeutic guidelines [235,454]. The current lack of reliable biomarkers to predict which patients will benefit from autophagy modulation also hampers the ability to stratify patients and optimize treatment outcomes [235,453]. More advanced animal models are also needed to study the role of specific autophagy-associated genes in tumor progression and response to treatment, as current models often fail to replicate the complexity of human cancer [235].

#### 8.2.2. Clinical Implications

Clinically, the balance between the inhibition of autophagy to target cancer cells and the minimization of toxicity to normal tissues remains a considerable challenge. In cancer treatment, it is critical to identify the therapeutic window that maximizes efficacy while minimizing side effects [453,454]. Increased autophagy during chemotherapy has been shown to contribute to drug resistance in cancer, leading to disease recurrence. Understanding this phenomenon is essential to overcome treatment failure and improve patient outcomes [129,235]. Tumor heterogeneity also complicates treatment, as the extent of autophagy dependency differs between cancer types and stages, making a one-size-fits-all approach difficult [235]. The complexity of combination therapies, particularly the integration of autophagy modulators with conventional or targeted therapies, also requires extensive research to determine the most effective treatment programs [235,453]. The development of selective inhibitors that specifically target autophagy in cancer cells without affecting normal cells is still a major challenge due to the risk of off-target effects [453,454].

Despite promising preclinical findings, robust clinical evidence supporting the efficacy of antioxidants in cancer therapy is limited. Many studies are underpowered or fail to address the complex interactions between antioxidants, cancer cells, and chemotherapy, highlighting the need for large-scale controlled trials to establish clear guidelines [455]. While antioxidants may improve the tolerability of chemotherapy by reducing side effects, careful evaluation of their interactions with chemotherapeutic agents is needed to avoid compromising treatment outcomes [456].

### 8.3. Emerging Technologies and Approaches

Emerging technologies and approaches in cancer therapies related to autophagy and oxidative stress encompass several key areas. Targeted autophagy modulation focuses on developing selective inhibitors that target autophagy in cancer cells while sparing normal tissues, reducing systemic toxicity and enhancing the efficacy of conventional treatments such as chemotherapy and radiotherapy [137]. Oxidative stress manipulation involves strategies to selectively increase ROS production in tumor cells or inhibit antioxidant pathways like those regulated by sirtuin 3 (Sirt3), thereby sensitizing cancer cells to ROS-induced cytotoxicity while minimizing effects on normal tissues [235]. Additionally, iron homeostasis targeting leverages the role of autophagy in regulating intracellular iron levels to disrupt tumor survival and proliferation [457].

Autophagy-based immunotherapies explore the modulation of autophagy in immune cells, such as dendritic cells and T lymphocytes, to improve anti-tumor immune responses [453]. Identifying autophagic biomarkers is another critical focus, with efforts aimed at discovering markers from human biopsy samples to stratify cancer subtypes and guide autophagy-inhibiting therapies [453]. Similarly, metabolic therapies target the interplay between autophagy and tumor metabolism, such as glutaminolysis, to exploit cancer cells’ metabolic vulnerabilities, reduce resistance, and enhance treatment efficacy [453].

The integration of experimental methodologies and biocomputational techniques plays a pivotal role in advancing these therapeutic strategies. Experimental approaches include genetic modulation, biomarker identification, metabolic profiling, immunomodulation, and the use of nanoparticle delivery systems to enhance precision and reduce off-target effects [458,459]. In contrast, biocomputational techniques utilize machine learning, network analysis, and systems biology to predict drug responses, identify therapeutic targets, and optimize treatment strategies. High-throughput screening, pathway analysis, and pharmacogenomics further facilitate personalized medicine approaches, enabling the rational design of drug combinations that integrate autophagy inhibitors with chemotherapy or targeted therapies for maximum therapeutic benefit [460]. These multidisciplinary advancements are reshaping cancer treatment paradigms by exploiting the dynamic interplay between autophagy and oxidative stress.

## 9. Conclusions

Autophagy and oxidative stress are essential mechanisms for maintaining cellular homeostasis, and their intricate interplay plays a pivotal role in cancer biology by influencing tumor progression, metastasis, and therapy resistance. Over the last decade, numerous studies have demonstrated that autophagy can act as both a tumor suppressor and a pro-tumorigenic mechanism, depending on the cancer type, stage, and microenvironment. In addition, autophagy promotes the survival of cancer cells under stress conditions such as hypoxia and nutrient deprivation.

This review highlights the dual role of autophagy and reactive oxygen species (ROS) in mediating cancer cell death and suppressing tumor progression in hematological malignancies. This interplay is tightly regulated by key signaling pathways, including PI3K/AKT/mTOR, AMPK, and HIF-1α, which maintain a balance between autophagic activity and ROS production. Notably, the dysregulation of autophagy can paradoxically exacerbate oxidative stress, establishing a feedback loop that promotes tumor survival and growth.

Understanding the crosstalk between autophagy and oxidative stress in tumorigenesis offers promising opportunities for targeted cancer therapies. Strategies such as autophagy inhibition, the amplification of ROS levels using pro-oxidant compounds, and the integration of these approaches with conventional treatments have shown potential to overcome therapeutic resistance and improve clinical outcomes. However, effective clinical translation requires a nuanced understanding of tumor-specific contexts and the dynamic nature of the autophagy–oxidative stress axis. This review underscores the need for continued research to refine therapeutic strategies and leverage this interplay for more effective and personalized cancer treatments.

## Figures and Tables

**Table 1:** Reactive species classification.

Classification	Definition	Species	Abbreviations
Free Radicals	At least one unpaired electron, making them highly reactive.	Superoxide	O_2_^−^
		Hydroxyl radical	HO·
		Peroxyl radical	ROO·
		Alkoxyl radical	RO·
		Nitric oxide *	NO·
Non-radicals	Reactive species without unpaired electrons but, still, participating in oxidative reactions. They can form radicals under certain conditions.	Hydrogen peroxide	H_2_O_2_
		Singlet oxygen	^1^O_2_
		Hypochlorous acid *	HOCl
		Ozone	O_3_
		Organic hydroperoxides	ROOH

The term reactive oxygen species (ROS) is frequently employed to refer to reactive oxygen-containing molecules, as well as reactive nitrogen or chlorine species. * These chemical species are examples of reactive nitrogen and chlorine species.

**Table 2:** Antioxidant defense systems.

Classification	Antioxidant	Characteristics
First-line	Superoxide dismutase (SOD)	Endogenous; enzymatic. Degradation of superoxide anions to more stable ROS: O_2_^−^ → H_2_O_2_ Three isoforms: cytoplasmic Cu/Zn-SOD (SOD1); mitochondrial Mn-SOD (SOD2), and EC-SOD (SOD3).
	Catalase (CAT)	Endogenous; enzymatic. Abundant in peroxisomes, it is absent in mitochondria of mammalian cells. Degradation of hydrogen peroxide O_2_^−^ → H_2_O_2_ → H_2_O + O_2_
	Glutathione peroxidase (GPX)	Endogenous; enzymatic. Mainly expressed in the mitochondria and sometimes in the cytosol. Degradation of hydrogen peroxide, with glutathione as substrate: O_2_^−^ → H_2_O_2_ → H_2_O + O_2_ GSH → GSSG Its activity may depend on its cofactor selenium, so it is known as selenocysteine peroxidase.
Second-line	Thioredoxin (TRX) system	Endogenous; first- or second-line defense depending on the author. Antioxidant proteins that facilitate reduction in proteins by cysteine thiol-disulfide exchange.
	Glutathione (GSH)	Endogenous; non-enzymatic first- or second-line defense depending on the author. Cofactor for GPx; directly neutralizes free radicals and ROS.
	Coenzyme Q10	Endogenous ubiquinone or exogenous from diet; non-enzymatic. Participates in the ETC and neutralizes free radicals within mitochondria.
	Carotenoids	Exogenous; non-enzymatic. Efficient quench of singlet oxygen and upregulation of antioxidant enzyme activity.
	Flavonoids	Exogenous; non-enzymatic. Direct free radical scavengers and metal-chelating properties.
	Vitamin C	Exogenous; non-enzymatic. Ascorbate enters cells from plasma by co-transporters, being particularly effective at scavenging superoxide radicals where SOD activity is lower.
Third-line	Nrf2 Autophagy Mitophagy	Endogenous adaptive response. It involves all mechanisms that upregulate antioxidant systems to remove free radicals left during the previous lines of defense.
